# A comparative survey of functional evidence use in hearing and vision loss genetics

**DOI:** 10.1038/s43856-026-01650-2

**Published:** 2026-05-30

**Authors:** R. Arda Inan, Marina T. DiStefano, Sami S. Amr, Tim Beißbarth, Lea M. Starita, Andrew B. Stergachis, Ahmad Abou Tayoun, Robert B. Hufnagel, Barbara Vona

**Affiliations:** 1 Institute for Auditory Neuroscience and InnerEarLab, University Medical Center Göttingen, Göttingen, Germany; 2 The Broad Institute of MIT and Harvard, Cambridge, MA USA; 3 Broad Clinical Labs, The Broad Institute, Cambridge, MA USA; 4 Laboratory for Molecular Medicine, Mass General Brigham Personalized Medicine, Cambridge, MA USA; 5 Department of Medical Bioinformatics, University Medical Center Göttingen, Göttingen, Germany; 6 Campus Institute Data Science (CIDAS), University of Göttingen, Göttingen, Germany; 7 Department of Genome Sciences, University of Washington, Seattle, WA USA; 8 Brotman Baty Institute for Precision Medicine, Seattle, WA USA; 9 Division of Medical Genetics, Department of Medicine, University of Washington, Seattle, WA USA; 10 Genomics Medicine Center, Dubai Health, Dubai, United Arab Emirates; 11 Center for Genomic Discovery, Dubai Health, Mohammed Bin Rashid University, Dubai, United Arab Emirates; 12 Department of Genetics and Center for Integrated Healthcare Research, Kaiser Permanente Hawaii Region, Honolulu, HI USA; 13 Auditory Neuroscience and Optogenetics Laboratory, German Primate Center, Göttingen, Germany; 14 Collaborative Research Center 1690 (CRC1690), University of Göttingen, Göttingen, Germany; 15 Department of Obstetrics and Gynecology, Brigham and Women’s Hospital, Harvard Medical School, Boston, MA USA

**Keywords:** Hereditary eye disease, Medical genetics

## Abstract

**Background:**

Despite advances in sequencing and standardized guidelines, many variants, particularly in genetically heterogeneous disorders such as hearing and vision loss, remain of uncertain significance. Multiplexed assays of variant effects (MAVEs) offer a solution to resolve these uncertainties, but their clinical integration is limited. We aimed to assess the utilization of functional evidence in hearing and vision loss genetics.

**Methods:**

We surveyed 82 hearing and ocular genetics experts, collecting quantitative and qualitative data on their practices, confidence in functional assays and perceived barriers to integrating functional evidence into clinical workflows.

**Results:**

Here we show that hearing and vision loss experts frequently encounter variants of uncertain significance and note that functional data often fails to resolve uncertainty due to limited availability or inconsistent quality. Confidence is highest in transcript assays, patient-derived cell models and computational predictors, while familiarity and trust are lower for non-mammalian models and high-throughput assays like MAVEs. Respondents highlight the need for improved data accessibility, standardized evaluation guidelines and training to support functional evidence integration.

**Conclusions:**

Our survey identifies both common and domain-specific challenges in applying functional evidence for variant classification in hearing and ocular genetics, emphasizing the need for frameworks that account for biological complexity, methodological limitations, and barriers to data accessibility.

## Plain language summary

Genetic testing is widely used to diagnose hearing and vision loss, but many DNA changes cannot be classified as harmful or harmless. This uncertainty makes it difficult for doctors to give patients reliable answers. Laboratory approaches that test how genetic changes affect biological function may help, but their use in clinical practice is still limited. In this study, we surveyed 82 experts in hearing and vision genetics from 30 countries to understand how they use such laboratory data. We found that although functional data can help, it is often inconsistent, limited and/or difficult to interpret. These findings suggest that better access to high-quality data, clearer guidelines and improved training could help make genetic diagnoses more accurate and useful for patients.

## Abstract (web summary)

Inan et al. surveyed 82 hearing and vision genetics experts to assess how functional evidence is used in variant classification. Experts report frequent variants of uncertain significance, limited and inconsistent functional data, and a need for standardized guidelines, better data access, and training to improve clinical integration.

## Introduction

Sequencing remains crucial for first-line diagnostics in clinical genetics; however, evaluating the clinical impact of the vast number of identified sequence variants represents a significant challenge. Variant classification guidelines have been harmonized by the American College of Medical Genetics and Genomics (ACMG)/Association of Molecular Pathology (AMP)^1^, and refined by the Clinical Genome Resource (ClinGen)^2–6^, which established Variant and Gene Curation Expert Panels (VCEPs/GCEPs) to provide domain-specific specifications for clinical geneticists. However, despite advances in classification guidelines and the growing availability of genomic and clinical data, the clinical impact of many rare variants remains uncertain. As of July 2025, over 2 million variants, corresponding to 57% of ClinVar entries, are variants of uncertain significance (VUSs) or have conflicting interpretations of pathogenicity^7^. The high abundance of VUS reflects insufficient evidence available to determine clinical significance of these variants, limiting the effectiveness of genetic testing to inform clinical decision-making.

This challenge is particularly evident in genetically heterogeneous disorders such as hearing loss (HL) and vision loss (VL), which share substantial gene overlap. The Genomics England PanelApp lists 191 HL- and 518 VL-associated genes, 41 of which are shared across both panels, as curated by experts^8^. Despite extensive submissions to ClinVar, the burden of VUS remains high, with 44% of HL variants and 46% of VL variants currently classified as VUS or having conflicting classifications across different clinical labs. The Deafness Variation Database (DVD) further illustrates the scale of this issue, classifying 89% of variants across 224 deafness-related genes designated as VUS^9^. This VUS prevalence is also reflected in the diagnostic rates of clinical reports, with positive diagnosis in large-scale studies of inherited hearing^10,11^ and ocular diseases^12,13^, currently between 39 and 51%. These figures collectively emphasize a challenge that is expected to grow as genomic testing becomes more widespread, underscoring the need for improved variant classification strategies to reduce uncertainty and improve diagnostic accuracy across neurosensory genetic conditions, which affect a substantial proportion of the population.

Multiplexed assays of variant effects (MAVEs) represent a promising, high-throughput avenue for resolving VUS by providing large-scale quantitative functional data in relevant cellular contexts. The field is actively shifting toward proactively creating comprehensive “atlases of variant effects” to reduce uncertainty and enable more accurate variant interpretation^14^. Notably, recent functional studies for *CNGA3*^15^ and *KCNQ4*^16^ have demonstrated translational utility of medium- and high-throughput assays by contributing to the reclassification of many VUS associated with VL and HL, respectively. While ongoing efforts aim to enable the reclassification of most missense VUSs by 2030^17^, the integration of MAVE data into routine clinical interpretation remains limited, with reports suggesting unclear levels of familiarity, preparedness, and willingness to use it among clinical genetics specialists^18^.

A recent survey by Park et al.^19^ revealed general challenges faced by genetics professionals regarding the availability, quality, and interpretation of functional data across various fields. Building on these findings, our study focuses specifically on hearing and ocular genetics experts to assess their readiness, expectations, and barriers to adopting functional data in clinical variant classification. We aim to illuminate domain-specific needs and opportunities for integrating functional evidence into specialized diagnostic workflows by concentrating on the common ground shared by these neurosensory fields.

To this end, we conducted a structured survey and received responses from 82 experts from hearing and/or ocular genetics, engaging members of the ClinGen HL VCEP and Ocular Clinical Domain Working Groups. Our goal was to elucidate current challenges in accessing, interpreting, and applying variant-level functional data, particularly in relation to VUS resolution. Specifically, we assessed the confidence of respondents in applying the ACMG/AMP Guidelines^1^ and ClinGen specifications for PS3 and BS3^3^, defined as well-established in vitro or in vivo functional studies showing a damaging or neutral effect on the gene or gene product, as well as domain-specific VCEP guidelines to evaluate functional evidence.

In our study, experts in HL and VL genetics report that VUS are common in routine practice, while functional evidence currently resolves only a limited proportion. Although confidence varies by evidence type, awareness in MAVEs is limited among respondents, with notable regional differences. Across functional approaches, low confidence in accuracy and limited access to information on data quality are major barriers to clinical use. By fostering cross-domain collaboration, this study seeks to inform future efforts to create resources, guidelines, and collaborations that support the effective use of functional data. Lessons from this effort may also inform broader initiatives to standardize functional evidence interpretation and improve diagnostic yield across rare disease domains.

## Methods

### Survey design and distribution

This survey was inspired by Park et al.^19^ but uniquely focused on assessing practices and challenges within the hearing and ocular genetics domains. The questions were carefully developed through a consensus-driven process involving domain experts (A.A.T., S.S.A., M.T.D., R.B.H., and B.V.) via a series of exchanges by email and video conferences. The survey was hosted on a publicly accessible REDCap platform^20^ and was open for a period of six months. It was distributed internationally using professional networks of the study team, collecting contacts on PubMed from researching publications reporting on the sequencing of hearing and ocular genetics patient cohorts, through promotion via multiple channels including the ClinGen Resource Quarterly Update, ACMG calls for collaboration, circulation via HL VCEP and Ocular GCEP email lists, Genomics England PanelApp reviewers for hearing loss, auditory neuropathy spectrum disorder, ophthalmological ciliopathies and retinal disorders, social media outreach, and websites or newsletters of affiliated institutions and genetics societies, such as the European Society of Human Genetics. Participation was voluntary, anonymous, and without any direct incentives.

### Survey structure

The survey was conducted in English and consisted of 62 items, including multiple-choice, Likert scale, and open-ended questions on: (1) demographic information, (2) current and future professional activities, (3) scope and challenges of variant interpretation, (4) functional evidence-related tasks, (5) confidence using functional evidence, (6) use of resources that include functional evidence, (7) use of guidelines for application of functional evidence, (8) challenges in utilizing functional evidence, (9) approaches to improving the interaction and application of functional evidence, (10) assessment of existing data on improving clinical utility, (11) use and expectations of gene-level functional data, and (12) use and expectations of variant-level functional data. A complete copy of the survey is provided in the Supplementary Document.

### Survey analysis and visualization

A total of 198 individuals began the survey, resulting in 82 complete responses. While no formal exclusion criteria were applied, individuals who were not actively participating in clinical variant interpretation for either HL or VL were prompted to discontinue the survey. One respondent filled in the physical version of the survey. Their answers were transferred to the REDCap database by the authors. Another respondent who only responded to questions related to demographics and professional activities was excluded from further analyses. REDCap data were securely stored on the servers of the Society for Scientific Data Processing Göttingen.

### Statistics and reproducibility

All quantitative analyses and visualizations were performed using R (version 4.4.0) with packages including data.table, dplyr, ggpubr, ggplot2, patchwork, reshape2, rnaturalearth, showtext, tidyr, and writexl. Median Likert scores were calculated to identify differences across expert groups. For assessment of differences in engagement with functional data across geographic regions, the analysis was restricted to continents and countries with at least 10 respondents. Geographical differences were assessed using the Kruskal-Wallis test, with pairwise comparisons performed using the Wilcoxon rank-sum test. Open-ended answers related to handling conflicting functional evidence (*n* = 37) were analyzed for common thematic elements after filtering out irrelevant answers. Responses to other open-ended questions were discussed contextually within the text where relevant.

### Ethics declaration

This study was conducted in accordance with the principles of the Declaration of Helsinki. The study protocol was reviewed and approved by the Ethics Committee of the University Medical Center Göttingen under protocol number 12/8/24 An. All data were fully anonymized at intake.

## Results

### Participant demographics, professional focus and experiences with VUS

We received 82 complete responses to the survey, following direct email contact with 477 participants and broad promotion across professional channels, representing 30 countries, with the highest participation from the United States of America (16%) and Germany (13%) (Supplementary Fig. 1A). The majority (73%) worked in academic medical centers and 41% of respondents were affiliated with ClinGen, with varying degrees of experience in variant classification (Supplementary Fig. 1B, C). Participants were divided into three main expert groups: 48% (39/82) specialized in hearing loss (HL), 23% (19/82) in vision loss (VL), and 29% (24/82) had cross-disciplinary expertise (H + V). Clinical roles were slightly more prevalent than research roles among HL experts (49% vs. 31%), whereas research roles predominated among VL experts (52% vs. 37%) (Supplementary Table). The respondents represent a diverse and multidisciplinary group, ensuring broad perspectives on variant interpretation in the fields of hearing and ocular disorders.

Experts across both domains report a high volume of variants, with about half curating over 100 variants annually (Supplementary Fig. 2A). Many variants are classified as VUS due to insufficient data and functional evidence resolves only a subset of these cases (Supplementary Fig. 2B, C). Despite this, most respondents reclassify 1–10 variants each year, but only a subset is driven by new data (Supplementary Fig. 2D, E). Conflicting functional evidence is a significant, common challenge, reported by 54% of participants (Supplementary Fig. 1D). Strategies for resolving conflicts include independent assessment of experimental design, adjusting the application of PS3/BS3 criteria, consulting peers, comparing with other evidence, performing additional experiments or following ClinGen guidance (Table 1). Taken together, it is suggested that functional evidence can aid variant classification, but barriers, including conflicting results and limited data, persist.

**Table 1:** Approaches to handling conflicting functional evidence

Assess the experiment (*n* = 10)	“I am reviewing each functional experiment (material and methods) to conclude which is the strongest evidence.” “Assessment of the functional studies by considering the model organism(s) utilized, assay calibration, usage of negative and positive controls, type of experiments performed, impact factor of the publishing journal.” “I have weighed the corresponding papers, and made a calculated decision based on my own interpretation of the results (validity, specificity), taken together with the type of journal, the authors, etc.”
Modify the guidelines (*n* = 7)	“Classified variant as VUS unless obvious problem with the data.” “Not applying either PS3 or BS3 at any level strength.” “Either don’t use functional evidence at all, or modify PS3/BS3 applicable.”
Discuss with peers (*n* = 4)	“The practice in ClinGen VCEPs is to consult the experts about which piece of functional evidence is more convincing, if any.”
Compare with other evidence (*n* = 3)	“I integrate with other evidence and consider clinical, segregation, and computational data to support or refute functional findings.”
Perform further experiments (*n* = 3)	“As I am lucky to work in a research genetic lab myself, I performed a new functional validation and confirmed one of the previous assessments.”
Follow ClinGen specifications (*n* = 2)	“As part of a ClinGen VCEP, (…) if results from different assays are conflicting for a single variant, then the result from the assay with the highest level of validation and a conclusive result can override the result from the other assay.”

*n* number of respondents/mentions, *VUS* variant of uncertain significance, *VCEP* variant curation expert panel, *PS3* pathogenic strong 3 (ACMG/AMP functional evidence criterion supporting pathogenicity), *BS3* benign strong 3 (ACMG/AMP functional evidence criterion supporting benign impact).

### Professional roles and collaborative engagement

**Fig. 1:**
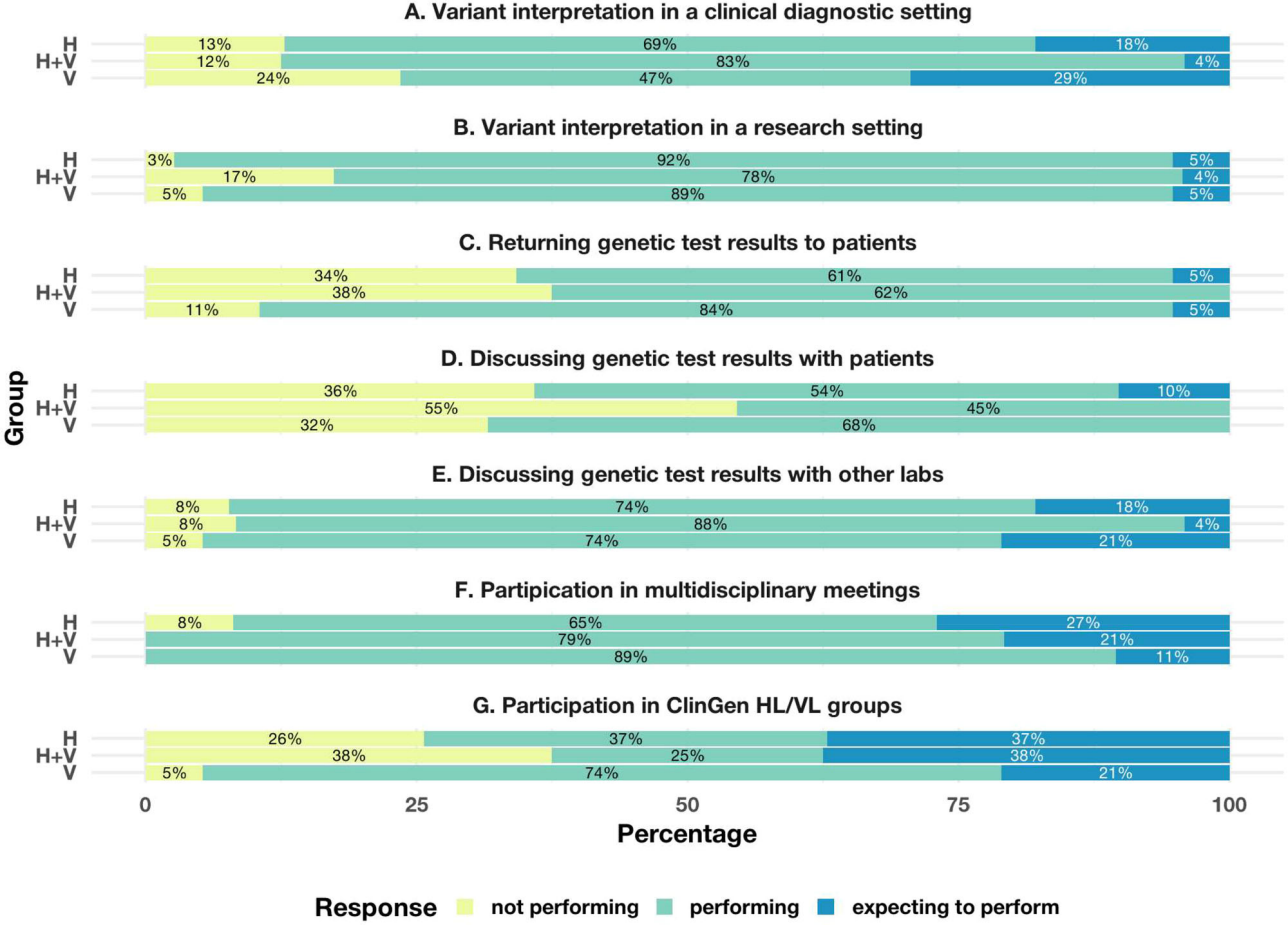
Current and anticipated professional activities. Survey respondents (H hearing, V vision, H + V both hearing and vision) were asked about their current and expected participation in variant interpretation and related professional activities. Bars indicate the proportion of experts within each domain who reported not performing (yellow), currently performing (teal), or expecting to perform (blue) each activity (**A**–**G**).

We explored the professional roles of HL, VL, and combined H + V experts by asking about their participation in clinical and research activities. Most HL (69%) and H + V specialists (83%) perform variant classification in clinical settings, compared with 47% of VL experts, likely reflecting the higher proportion of research-focused respondents in the VL group (Fig. 1A). Research-based variant classification, discussing genetic test results with other labs and participating in multidisciplinary meetings were common across all groups (65–92%) (Fig. 1B, E). Notably, VL experts demonstrated a broader clinical communication role, more frequently reporting that they communicate results directly with patients (68–84% vs. 45–62%) (Fig. 1C, D, F). VL specialists also show higher current involvement in ClinGen Working Groups (74% vs. 25–37%) (Fig. 1G). These results suggest that HL, VL, and H + V specialists participate actively in research, variant classification, multidisciplinary discussions and collaborative efforts, underscoring their common commitment to improving the interpretation and application of genetic data.

### Engagement with functional evidence

Functional evidence can be broadly categorized into diagnostic-grade and research-grade evidence. Diagnostic-grade evidence refers to assays that are sufficiently validated, reproducible, and well-calibrated to support variant interpretation within ACMG/AMP frameworks. To gain insight into how HL and VL professionals engage with functional evidence, we asked respondents which functional evidence-related tasks they are currently performing. Across all groups, most respondents reported using public data sources such as ClinVar^7^, MaveDB^14^ and PubMed^21^ (58–87%), reassessing previously classified variants by reviewing newly available functional evidence (68–83%) and curating the validity and relevance of functional assays for variant classification (58–67%) (Supplementary Fig. 3A–C). However, requests for diagnostic-grade functional evidence from clinical laboratories were relatively uncommon (37–45% vs. 43–74%) compared to research-grade evidence, likely reflecting the limited availability of approved clinical assays for both HL- and VL-associated genes (Supplementary Fig. 3D, E). While most European experts reported sourcing research-grade functional evidence directly from laboratory studies, this practice was not widespread in the United States (Supplementary Fig. 4A). Collaboration with research laboratories was common across domains, including those specializing in hearing or vision loss (57–63%) or in fundamental mechanisms and protein properties (38–51%) (Supplementary Fig. 3F, G). Overall, HL and VL specialists actively engage with functional evidence through public resources and collaborations, with an emerging interest in expanding functional data use.

### Confidence with functional evidence types and models

**Fig. 2:**
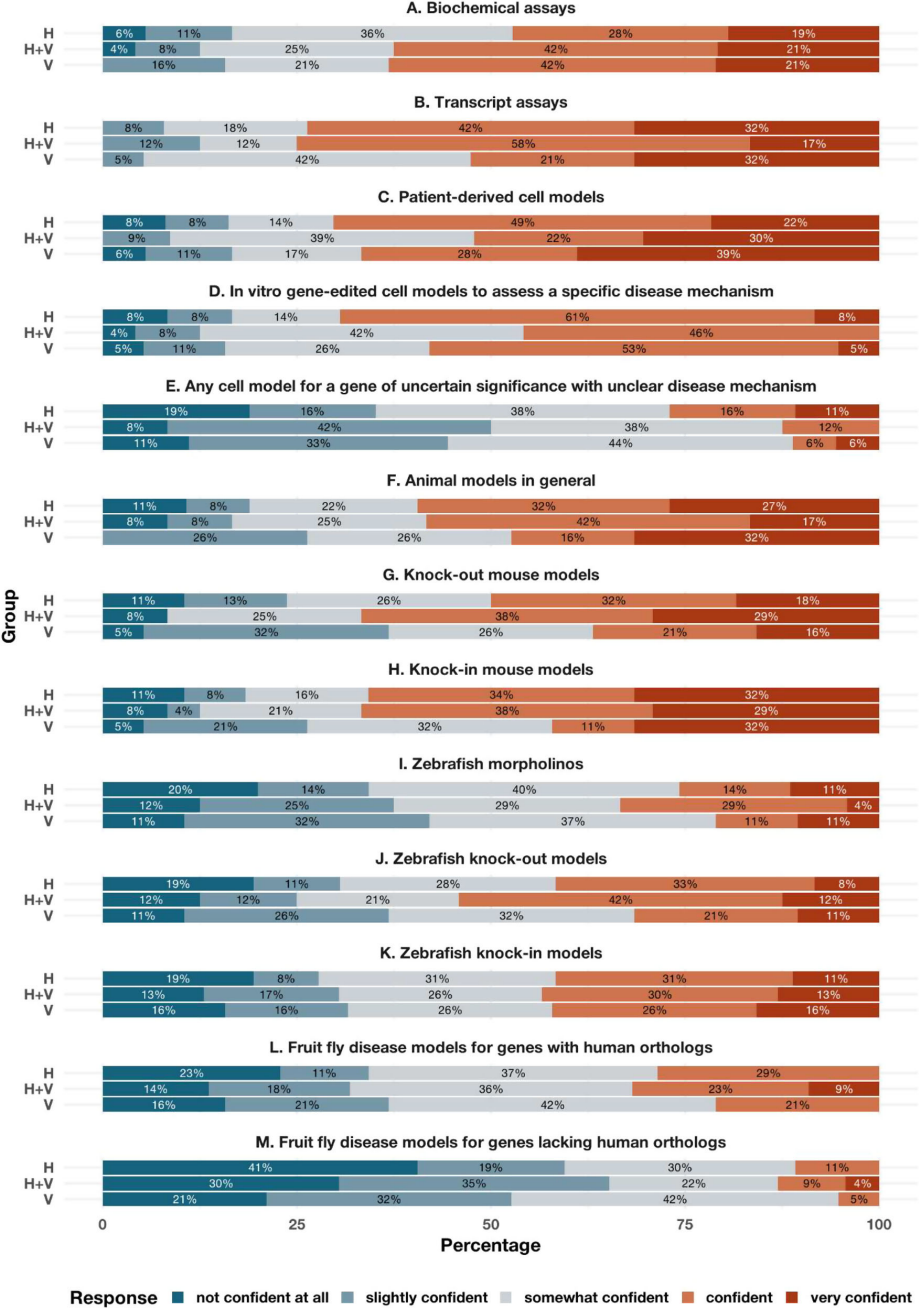
Confidence in using functional evidence. Stacked bar plots display confidence levels reported in applying various types of functional assays for variant interpretation (**A**–**M**) across disease domains: hearing loss (H), ocular diseases (V), and both domains (H + V). Responses were collected on a five-point Likert scale and grouped into three percentage categories: (i) not confident/slightly confident (blue), (ii) somewhat confident (gray), and (iii) confident/very confident (red). Missing responses (1–6 per item) were not shown.

Respondents rated their confidence in applying different functional data types for variant classification, from *not confident at all* to *very confident*. Experts expressed moderate confidence in biochemical assays (47–63%) (Fig. 2A). Transcript-based assays, such as splicing assays, (53–75%) and patient-derived cell models (52–70%), were generally trusted across all respondents (Fig. 2B, C). Confidence in gene-edited in vitro models ranged from 46 to 69% (Fig. 2D). Complex in vivo systems, including various animal models, were trusted by 37–67% of respondents (Fig. 2F–H), while less complex models, such as cell models for genes of uncertain significance, zebrafish, and *Drosophila* models, were met with moderate to low confidence across all groups (11–54%) (Fig. 2E, I–M). These results indicate that respondents had highest confidence in biochemical and transcript-based assays, moderate confidence in gene-edited in vitro models and more variable confidence in complex in vivo systems.

### Familiarity and confidence in functional evidence resources

**Fig. 3:**
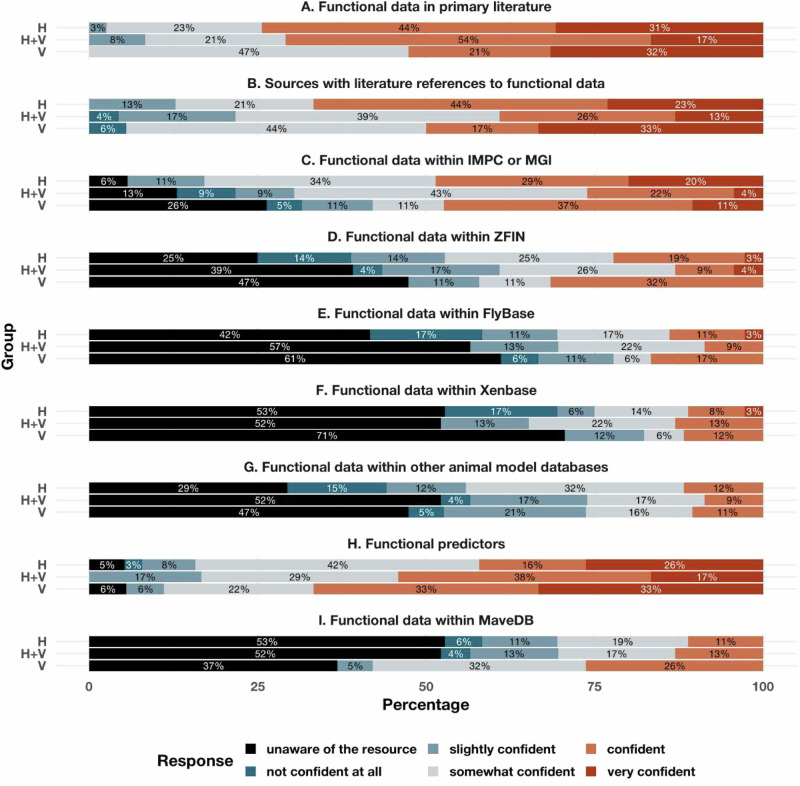
Confidence in functional evidence resources. Stacked bar plots display confidence levels reported in functional evidence resources (**A**–**I**) across disease domains: hearing loss (H), ocular diseases (V), and both domains (H + V). Responses were collected on a six-point Likert scale and grouped into three percentage categories: (i) not confident/slightly confident (black/blue), (ii) somewhat confident (gray), and (iii) confident/very confident (red). Missing responses (1–6 per item) were not shown.

Experts rated their confidence in utilizing a variety of available functional evidence resources. The highest confidence was consistently reported for functional data found in the primary literature and established literature-based clinical databases such as ClinVar^7^, LitVar2^22^, and OMIM^23^ (39–74%) (Fig. 3A, B). Mouse model databases were perceived with moderate confidence (26–49%) (Fig. 3C), non-mammalian resources (e.g., ZFIN^24^, FlyBase^25^, Xenbase^26^) were generally unfamiliar or unreliable (9–32%) (Fig. 3D–G). Computational predictors were viewed with moderate to high confidence (42–67%) (Fig. 3H). Although overall trust in MaveDB^14^ was limited across all domains (11–26%) (Fig. 3I), the trend varied geographically as a majority of respondents from Europe (66%) and Asia (53%) were unaware of this resource (Supplementary Data 2, Supplementary Fig. 4B). On the other hand, 62% of respondents from the United States reported confidence in MaveDB. Broadly, these insights suggest that experts have highest confidence in published and literature-curated sources for functional data and computational predictors, while confidence in non-mammalian functional data and high-throughput experimental assay data was comparatively limited.

### Confidence in standardized criteria

Confidence in applying PS3/BS3 criteria was assessed by their comfort level with relevant guidelines. Relatively high confidence (50–89%) was reported across the board for the ACMG/AMP Guidelines, ClinGen PS3/BS3 specifications, ClinGen splicing evidence recommendations and the ClinGen functional assessment worksheet (Supplementary Fig. 5A–C, E). Both HL and VL experts found domain-specific guidelines moderately useful (38–68%) (Supplementary Fig. 5D, F–I). However, confidence in the ClinGen glaucoma guidelines was lower among VL specialists (37%), with some unaware of these recommendations (Supplementary Fig. 5J). This discrepancy likely reflects the relative timing of guideline development, as the ocular domain guidelines were established more recently. In open-ended responses, one participant reported using X-linked inherited retinal disease specifications for *RPGR* and *RS1* in addition to the listed guidelines in this survey. Generally, specialists in HL and VL show broad familiarity with functional evidence guidelines.

### Improving and assessing the functional evidence application

#### Barriers to functional evidence application

Respondents rated the extent to which various barriers impact their use of functional data. Insufficient training, both general and disease-specific, was reported as an occasional challenge (21–42%) (Supplementary Fig. 6A–E). The most frequently reported challenge among HL experts was interpreting reduced penetrance or variable expressivity (58%) (Supplementary Fig. 6F). Concerns regarding the quality and accuracy of functional evidence were frequently reported (50–68%) (Supplemental Fig. 6G, H), as were challenges in locating and interpreting functional data in literature and variant databases (17–53%) (Supplementary Fig. 6I–L). Understanding clinically relevant transcripts for HL and VL-related genes also presented a moderate challenge (31–42%) (Supplementary Fig. 6M). Additional challenges cited by respondents in an open-ended section included the substantial time and effort required to evaluate assay validity and relevance, variability in guideline application, limited genetics knowledge among clinicians, and restricted access to publications.

#### Strategies for improvement

To understand how the use of functional evidence could be enhanced, respondents rated the impact of various factors. Workshops, additional VCEP specifications and disease-specific guidelines were widely regarded as useful (54–83%) (Supplementary Fig. 7A, E–G). Online training modules and training spreadsheets were rated as helpful (42–69%) (Supplementary Fig. 7B–D). Improved access to primary functional data and standardized assessments through ClinVar was viewed as highly beneficial (72–96%) (Supplementary Fig. 7H, I). Furthermore, the ability to request the de novo generation of functional data for specific variants or access gene-wide deep mutational scans was consistently rated as impactful for enhancing variant classification (58–86%) (Supplemental Fig. 7J, K).

### Expectations about clinical utility and future data use

#### Clinical utility and data curation needs

**Fig. 4:**
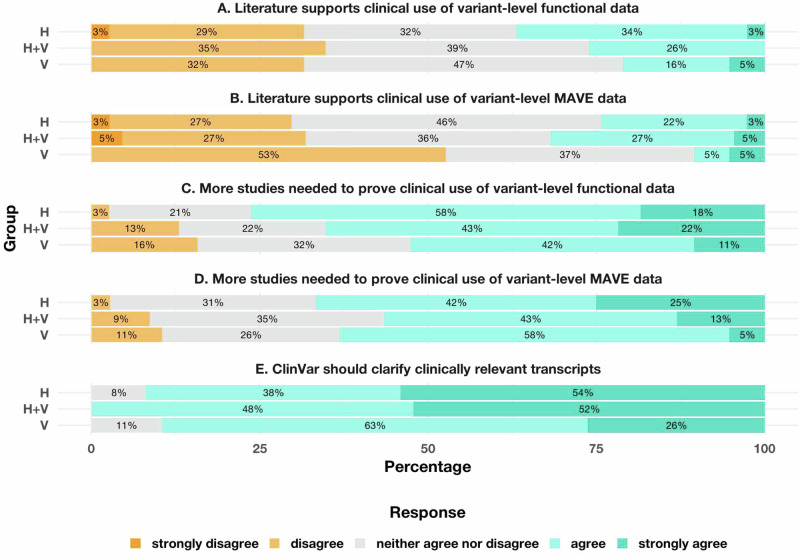
Assessment of existing data on improving clinical utility. Stacked bar plots display agreement with statements regarding interaction with functional assays to improve clinical utility (**A**–**E**) across disease domains: hearing loss (H), ocular diseases (V), and both domains (H + V). Responses were collected on a five-point Likert scale, ranging from strongly disagree (orange) to strongly agree (cyan), and grouped into two percentage categories: (i) strongly disagree/disagree, and (ii) agree/strongly agree. Missing responses (2–4 per item) were not shown. Neutral responses are excluded from display.

Respondents indicated their level of agreement with several statements regarding the role of functional evidence in clinical interpretation. Opinions were mixed on whether existing literature provides sufficient support for the clinical utility of variant-level functional data or MAVE datasets (Fig. 4A, B). Across all groups, there was a consensus on the need for additional studies demonstrating clinical utility and for clarifying clinically relevant transcripts in ClinVar (53–100%) (Fig. 4C–E).

#### Future expectations for functional data integration

HL and VL experts were asked about their current practices and future expectations regarding functional data use. They reported reviewing literature for both gene- and variant-level functional evidence during variant classification (78–91%) (Supplementary Figs. 8A and 9A). Gene-level data were considered highly helpful (74–87%) in the absence of variant-specific data (Supplementary Fig. 8B, C). Experts identified essential requirements for the proper use of variant-level functional data in ClinVar, including the specification of detailed methods as well as disease mechanism (65–95%) (Supplementary Fig. 9B, C). Metrics reflecting assay credibility (e.g., reproducibility, assay type, quality control measures), linked directly to primary data sources and by showing multiple resources, even if they conflict, were seen as beneficial (61–100%) (Supplementary Fig. 9D–G). A majority stated they would adopt pre-defined functional evidence levels (e.g., PS3_Strong or PS3_Supporting) if integrated into ClinVar (53–85%) (Supplementary Fig. 9H).

## Discussion

We captured the perspectives of 82 professionals on the use of functional evidence for variant classification for HL and VL, representing, to the best of our knowledge, the first survey focused on non-cancer hereditary disorders. Although functional evidence is expected to play a central role in resolving VUS, challenges remain in accessing high-quality functional data, interpreting complex methodological details, and integrating these findings into clinical decisions. Our findings emphasize the ongoing need for improved data transparency and structured evidence curation to strengthen variant classification practices.

HL and VL specialists demonstrated contrasting comfort levels with different types of functional assays. HL specialists were generally more hesitant to use biochemical assays for variant classification; this is likely reflecting a perceived lack of reliable and physiologically relevant assay systems for many HL-associated genes. This may stem from the limited availability of physiologically relevant assay systems and the stringent specifications adopted by HL Clinical Domain Working Group (CDWG). The ClinGen HL CDWG reserves PS3_Strong for knock-in mouse models, only specific biochemical assay types would achieve PS3 evidence. For example, for *GJB2*, electrical coupling and dye transfer assays were considered to provide moderate-level evidence^27^. On the other hand, the Ocular CDWG approved a broader range of in vitro functional assays as strong-level evidence for genes implicated in glaucoma, Leber Congenital Amaurosis/early onset Retinal Dystrophy and X-linked Inherited Retinal Disorders (IRDs). Given the anatomical and physiological differences between rodent and human ocular structures^28^, it is not surprising that VL experts put a greater emphasis on in vitro assays and computational predictors than in vivo models. Furthermore, HL and VL experts jointly expressed less trust in large-scale functional studies and corresponding functional evidence frameworks. Such tendency may reflect the limited knowledge of the molecular mechanisms of many HL- and VL-associated genes, as functional assays might not account for the full biological complexity (e.g., hair cell biology) that may be impacted. Therefore, it should be noted that functional evidence can be asymmetrical, since a damaging phenotype in a single assay does not rule out deleterious effects through mechanisms not captured by that assay^29^. Conversely, an in vitro or in vivo damaging effect may be rescued by a *cis*-acting variant masking the clinical effect^30^. Taken together, these findings indicate the need for disease-specific functional evidence frameworks that can better accommodate the distinct biological and methodological challenges of each domain.

HL experts specifically reported challenges related to the application of functional evidence when the possibility of reduced penetrance or variable expressivity exists. Several cases of incomplete penetrance and modifier effects have been previously reported for HL^31–34^. While large-scale functional studies like MAVEs are expected to help resolve many VUSs, they are not anticipated to resolve all VUS due to biological heterogeneity and mechanistic complexity caused by variable penetrance^35^. To address these limitations, groups such as the Cancer Variant Interpretation Group UK (CanVIG-UK) have proposed frameworks to interpret variants of reduced penetrance, particularly those manifesting intermediate or conflicting phenotypes in functional assays^36^. Given that gene regulatory variation can modify penetrance^37^, it is also important to integrate insights from high-throughput assays for regulatory variation, such as massively parallel reporter assays (MPRAs)^38^. Moreover, emerging guidelines specifically for interpreting non-coding variants may provide valuable insights into how these variants influence gene expression and disease risk^39^. These multifaceted approaches may offer a more comprehensive strategy for reducing uncertainty in variant classification by complementing findings from large-scale functional assays.

Considering that the term VUS encompasses variants with a broad range of posterior probabilities of pathogenicity (10–90%), subclassifying them could further refine clinical decision-making processes. Supporting this approach, a framework for VUS subclassification integrated by four clinical laboratories demonstrated the potential to substantially reduce uncertainty, with published data indicating that VUS sub-tiering resolved approximately 40% (23,067/57,480) of VUS in a large cohort^40^. Furthermore, it has been previously shown that HL- and IRD-specific guidelines improved variant classification, resolving 62%^41^ and 53%^42^ of VUS, respectively. These results strongly suggest that the revision of existing guidelines and integration of them with domain-specific recommendations can dramatically reduce uncertainty in variant classification.

The earlier survey of 190 genetics professionals^19^ and our survey of 82 domain experts in hearing and vision loss revealed several consistent themes. Both studies emphasize that limited availability, conflicting results, and uncertainty about assay validity are major barriers to incorporating functional data into variant interpretation. A pervasive lack of confidence in the quality and accuracy of available assays was identified as a major barrier, depicting the importance of resources like MaveMD (https://www.mavedb.org/mavemd), which integrates MAVE datasets with clinical evidence and structured outputs compatible with the ACMG/AMP guidelines. Participants in both studies expressed strong support for improved standardization, harmonized frameworks, and better access to primary functional data, as well as for integrating standardized PS3/BS3 interpretations into widely used resources such as ClinVar, to enhance the reliability and consistency of variant classification.

Our findings underline that both shared and disease-specific challenges exist in leveraging functional evidence for variant classification in HL and VL. While experts across both domains recognize the value of functional data, they identify barriers related to assay quality, lack of standardized assay systems, evidence evaluation criteria and limited data accessibility. Addressing these gaps through improved data sharing, methodological transparency, and specialized training will be essential to fully realize the clinical utility of functional evidence in variant classification. A future longitudinal study could help identify how the use of functional evidence evolves in response to new assays, updated evaluation criteria, and increased familiarity with available resources.

## Supplementary information

Transparent Peer Review file

Supplemental Material

Description of Additional Supplementary Files

Supplementary Data 1

Supplementary Data 2

## Data Availability

Variant data for VUS analysis in HL and VL genes were obtained from the most recent versions available at the time of access: ClinVar (July 21, 2025; https://www.ncbi.nlm.nih.gov/clinvar/docs/downloads/) and DVD v9.1 (January 4, 2021; https://deafnessvariationdatabase.org). Source data for all figures are provided in Supplementary Data 1.

## Code availability

The survey data and scripts used to generate the figures in this study are available through GitHub: https://github.com/ardainn/VUS-Hearing-Ocular/.
