# Supplementary material for: A comparative survey of functional evidence use in hearing and vision loss genetics
Source: Commun Med (Lond). 2026 May 30;6:343. doi: 10.1038/s43856-026-01650-2 (PMC13272955; doi:10.1038/s43856-026-01650-2)
Supplement: Supplementary file 2 — Supplemental Material [file 43856_2026_1650_MOESM2_ESM.pdf]

Supplementary Document

Assessment of Current and Future Approaches to Address Variants of Uncertain Significance in Hearing and Ocular Genomics Domains

Purpose of the QuestionnaireThis questionnaire aims to explore the practices and experiences of professionals in the hearing loss and ocular genetics domains who are actively incorporating variant-level functional data into workflows. By focusing on these areas, we seek to gather diverse perspectives on variants of uncertain significance (VUS) and better understand how current and future functional data will be integrated into clinical practice.We aim to address key topics, including: - The impact of VUS in routine workflows - The practical applications and challenges of using functional evidence criteria in variant classification - The level of confidence in applying functional evidence criteria - Expectations for the type of information included when aggregating variant classification evidence at a single source like ClinVar - Perspectives for future inclusion of high-throughput approaches, such as multiplexed assays of variant effect (MAVEs), into resources like ClinVar Our goal is to enhance understanding of the current awareness and application of functional data for interpreting VUS within these two clinical domains. We join international efforts to address the challenges posed by VUS. Functional data have conventionally been generated on a variant-by-variant basis using cell or animal models. However, scalable functional assays like MAVEs offer the potential to accelerate VUS reclassification, providing more certainty about whether variants are pathogenic or benign. This study seeks to capture current and future perspectives on these developments from active members of the hearing loss and ocular genetics communities. Estimated time for completion:15-20 minutes Ethics Approval:This study was approved by the Ethics Committee of the University Medical Center Göttingen (Approval: 12/8/24, Study Center ID: 2024-03475). Acknowledgement:We acknowledge Dr. Lea M. Starita and Dr. Andrew B. Stergachis from the Department of Genome Sciences, University of Washington, Seattle, WA, USA for inspiring this work with their preprint (https://doi.org/10.1101/2025.01.25.25321117). We appreciate your participation and willingness to contribute to this important work. Sincerely,Dr. Ahmad Abou TayounAl Jalila Genomics Center of Excellence, Al Jalila Children's Specialty Hospital, Dubai Health, Dubai, UAEDr. Sami S. AmrLaboratory for Molecular Medicine, Mass General Brigham Personalized Medicine, Cambridge, Massachusetts, USA Department of Pathology, Brigham and Women's Hospital, Boston, Massachusetts, USADeparment of Pathology, Harvard Medical School, Boston, Massachusetts, USADr. Marina DiStefanoMedical and Population Genetics, Broad Institute of MIT and Harvard, Cambridge, Massachusetts, USADr. Robert B. HufnagelCenter for Integrated Healthcare Research, Kaiser Permanente, Hawai'i Region, Honolulu, HI, USA Ophthalmic Genetics and Visual Function Branch, National Eye Institute, National Institutes of Health, Bethesda, MD, USADr. Barbara VonaInstitute for Auditory Neuroscience, University Medical Center Göttingen, Göttingen, Germany

Consent, Participation Eligibility, Data Use, and Contact Information

Consent We are conducting a study on the impact of variants of uncertain significance (VUS) and variant-level functional data. We are seeking insights and experiences from active hearing and ocular genetics professionals whose expertise are invaluable to our comprehensive understanding of the challenges and expectations of these communities.

Eligibility for participation We request that only individuals actively involved in clinical variant classification in hearing loss and/or retinal diseases participate in this questionnaire.

Participation is voluntary and anonymity is guaranteed
Participation is voluntary and your responses are anonymous. If you decide not to continue while completing the questionnaire, you can exit the page at any time to discontinue participation. By submitting the questionnaire, you consent to the use of your data. Please note that once submitted, responses cannot be deleted due to the anonymous nature of data collection.   Use of questionnaire data The anonymous data collected through this questionnaire will be used to explore current and future challenges faced by the hearing and ocular genetics communities. These data will be analyzed and contribute to the development of educational resources. Summarized responses may be published in peer-reviewed journals, included in educational materials, or be presented at conferences. All reponses are anonymous. We do not collect information that would identify participants. The questionnaire is estimated to take 15-20 minutes of your time. While participant perspective are vital in guiding the use of functional data in hearing and ocular genomic medicine and future development of databases, there is no immediate direct benefit for participating. However, the goal is that your participation will help improve the accessibility and utility of genomic databases, ultimately advancing scientific progress in hearing and ocular genomic medicine.

Contact information Please contact the study leader (Dr. Barbara Vona, barbara.vona@med.uni-goettingen.de) in case of questions or concerns.

Thank you for your participation!

Demographic Information

Select the disease area in which you are currently actively participating in clinical variant interpretation	◯ Hearing loss<br>◯ Vision loss<br>◯ Both<br>(If neither of these apply, please exit the survey)
What kind of organization do you work for? Select all that apply.	☐ Academic medical center<br>☐ Academic non-medical center<br>☐ Commercial laboratory<br>☐ Children's hospital<br>☐ Private practice<br>☐ Community hospital<br>☐ Government agency<br>☐ Pharmabiotech<br>☐ I'm retired<br>☐ Other
Are you in a leadership or managerial role?	◯ Yes<br>◯ No
What is your primary professional position?	◯ Lab director<br>◯ Lab technician<br>◯ Genetic counselor<br>◯ Clinical geneticist<br>◯ Laboratory medical geneticist<br>◯ Pathologist<br>◯ Molecular genetic pathologist<br>◯ Variant review scientist<br>◯ Research scientist<br>◯ Otolaryngologist<br>◯ Audiologist<br>◯ Ophthalmologist<br>◯ Other
How many years of professional experience do you have in your primary position?	◯ 0-5<br>◯ 6-10<br>◯ 11-15<br>◯ 16-20<br>◯ More than 20
What is the primary country in which you practice?	______________________
Do you perfom curations for any of the following organizations?	☐ ClinGen<br>☐ Genomics England<br>☐ Leiden Open Variation Database (LOVD)<br>☐ Orphanet<br>☐ The Gene Curation Coalition<br>☐ Other
Please list the organization	______________________
What is your gender?	______________________

Current and Future Professional ActivitiesWhich of the following activities do you perform now or anticipate performing in the future?

	I am not performing and will not perform in future	I am currently performing	I am not performing now but expect to in the future
Variant interpretation in a clinical diagnostic setting	○	○	○
Variant interpretation in a research setting	○	○	○
Returning genetic test results to patients	○	○	○
Discussing genetic test results with patients	○	○	○
Discussing genetic test results with other labs	○	○	○
Participation in multidisciplinary meetings (e.g. hearing or vision genetics meetings)	○	○	○
Participation in the ClinGen Hearing Loss Expert Panel or the ClinGen Ocular Clinical Domain Working Group, including any of the Variant Curation Expert Panels (VCEPs) or Gene Curation Expert Panels (GCEPs)	○	○	○

Scope and Challenges of Variant Interpretation

Question	Response
Do you participate in assessing genetic variants for potential clinical relevance, regardless of whether they are reported to a patient?	◯ Yes<br>(If "no", please exit the questionnaire)
In a typical year, approximately how many genetic variants do you evaluate for potential clinical significance, regardless of whether these assessments are reported to a patient?	◯ None<br>◯ 1-10<br>◯ 11-25<br>◯ 26-50<br>◯ 51-100<br>◯ More than 100
In a typical year, approximately what proportion of the variants that you classify as VUS are labeled as such due to insufficient data?	◯ None<br>◯ Some<br>◯ A moderate amount<br>◯ Many<br>◯ Very many
In a typical year, approximately what proportion of the variants that you classify as VUS have functional data available that you used for their classification?	◯ None<br>◯ Some<br>◯ A moderate amount<br>◯ Many<br>◯ Very many
In a typical year, approximately how many VUS do you reclassify based on new or updated data?	◯ None<br>◯ 1-10<br>◯ 11-25<br>◯ 26-50<br>◯ 51-100<br>◯ More than 100
In a typical year, approximately what proportion of VUS that you reclassified using new or updated data was specifically due to this new or updated data?	◯ None<br>◯ Some<br>◯ A moderate amount<br>◯ Many<br>◯ Very many
Have you encountered conflicting functional evidence or results while assessing functional evidence for variant classification?	◯ Yes<br>◯ No
Please describe how you have handled conflicting functional evidence:	__________________________________

Functional Evidence-Related TasksWhich functional evidence-related tasks are you currently performing or expect to perform in the future?

	I am not performing and will not perform in future	I am currently performing	I am not performing now but expect to in the future
Evaluate available functional data from public sources for research purposes	◯	◯	◯
Curate functional evidence for clinical variant classification	◯	◯	◯
Reassess variant classification in a clinical setting using functional evidence	◯	◯	◯
Request diagnostic-grade functional evidence from diagnostic labs	◯	◯	◯
Request research-grade functional evidence from research labs	◯	◯	◯
Request functional evidence from research labs specifically specialized in hearing and/or vision functional research, irrespective of diagnostic- or research-grade	◯	◯	◯
Request functional evidence from research labs specialized in the basic mechanism or protein structural or biophysical properties, not necessarily from the hearing or vision fields, irrespective of diagnostic- or research-grade	◯	◯	◯

Are there other activities involving functional evidence not listed above that you would like to add?

____

Confidence Using Functional EvidenceRate your confidence in applying the following types of functional evidence for genetic variant classification from 1 (not confident at all) to 5 (very confident), under the assumption of functioning experimental and clinical validation controls and sufficient replicates.

	1 (Not confident at all)	2	3 (Somewhat confident)	4	5 (Very confident)
Biochemical assays (e.g. enzymatic activity assays)	○	○	○	○	○
Transcript assays (e.g. splicing assays or transcriptomics data)	○	○	○	○	○
Patient-derived cell models	○	○	○	○	○
In vitro gene edited cell models to assess a specific disease mechanism (e.g. loss-of-function)	○	○	○	○	○
Any cell model for a gene of uncertain significance with unclear disease mechanism	○	○	○	○	○
Animal models in general (e.g. mouse or zebrafish models of genetic diseases)	○	○	○	○	○
Knock-out mouse models	○	○	○	○	○
Knock-in mouse models	○	○	○	○	○
Zebrafish morpholinos	○	○	○	○	○
Zebrafish knock-out models	○	○	○	○	○
Zebrafish knock-in models	○	○	○	○	○
Drosophila disease models for genes with human orthologs	○	○	○	○	○
Drosophila disease models for genes lacking human orthologs	○	○	○	○	○

Are there additional types of functional evidence beyond those mentioned above that you utilize in genetic variant classification? Please specify particular assay categories and explain their importance in providing functional data. ________________________

Use of Resources That Include Functional EvidenceRate your confidence in using the following functional evidence resources, on a scale of 1 (not confident at all) to 5 (very confident) or NA for signaling unawareness of the resource.

	NA - I am unaware of the resource	1 (Not confident at all)	2	3 (Somewhat confident)	4	5 (Very confident)
Functional data in primary literature	○	○	○	○	○	○
Sources with literature references to functional data (e.g. ClinVar, LitVar2, OMIM)	○	○	○	○	○	○
Functional data within the International Mouse Phenotyping Consortium (IMPC) or Mouse Genome Informatics (MGI)	○	○	○	○	○	○
Functional data within the Zebrafish Information Network (ZFIN) database	○	○	○	○	○	○
Functional data within FlyBase	○	○	○	○	○	○
Functional data within Xenbase	○	○	○	○	○	○
Functional data within other animal model databases	○	○	○	○	○	○
Functional predictors such as SpliceAI, AlphaMissense, and/or REVEL	○	○	○	○	○	○
Functional data within MaveDB	○	○	○	○	○	○

Use of Guidelines for Application of Functional EvidenceRate your confidence in using the following functional evidence guidelines for applying PS3/BS3 criteria, on a scale of 1 (not confident at all) to 5 (very confident) or NA for signaling unawareness of the guideline.

	NA - I am unaware of the guideline	1 (Not confident at all)	2	3 (Somewhat confident)	4	5 (Very confident)
ACMG/AMP guidelines (Richards et al., 2015)	○	○	○	○	○	○
ClinGen Sequence Variant Interpretation Working Group updated guidelines for functional evidence PS3/BS3 criteria (Brnich et al., 2020)	○	○	○	○	○	○
ClinGen Sequence Variant Interpretation Working Group updated guidelines for splicing evidence (Walker et al., 2023)	○	○	○	○	○	○
VCEP specific guidelines for functional evidence use	○	○	○	○	○	○
ClinGen Sequence Variant Interpretation Working Group functional assay assessment worksheet	○	○	○	○	○	○
Expert specification of the ACMG/AMP variant interpretation guidelines for genetic hearing loss (Oza et al., 2018)	○	○	○	○	○	○
ClinGen Hearing Loss Expert Panel Specifications to the ACMG/AMP Variant Interpretation Guidelines for CDH23, COCH, GJB2, KCNQ4, MYO6, MYO7A, SLC26A4, TECTA and USH2A Version 2.0	○	○	○	○	○	○
ClinGen Hearing Loss Expert Panel Specifications to the ACMG/AMP Variant Interpretation Guidelines for OTOF and MYO15A Version 1.0	○	○	○	○	○	○
ClinGen Leber Congenital Amaurosis/early onset Retinal Dystrophy Expert Panel Specifications to the ACMG/AMP Variant Interpretation Guidelines for RPE65 Version 1.0	○	○	○	○	○	○

ClinGen Glaucoma Expert Panel Specifications to the ACMG/AMP Variant Interpretation Guidelines for MYOC Version 1.1	○	○	○	○	○	○

Do you use other functional evidence guidelines for genetic variant interpretation that were not listed above? Please provide specific examples and explain their value. __________________________________

Challenges in Utilizing Functional EvidenceRate how the following barriers might prevent your use of functional evidence in variant classification on a scale of 1 (not a challenge for using functional evidence) to 5 (a significant challenge for using functional evidence).

	1 (Not a challenge for using functional evidence)	2	3 (Occasionally a challenge for using functional evidence)	4	5 (A significant challenge for using functional evidence)
Insufficient training on the general use of functional evidence for variant classification	○	○	○	○	○
Insufficient training on the use of functional evidence for variant classification of loss-of-function variants	○	○	○	○	○
Insufficient training on the use of functional evidence for variant classification of gain-of-function variants	○	○	○	○	○
Insufficient training on the use of functional evidence for variant classification of dominant negative variants	○	○	○	○	○
Insufficient training on the use of functional evidence for variant classification where haploinsufficiency is a possibility	○	○	○	○	○
Insufficient training on the use of functional evidence for variant classification where reduced penetrance or variable expressivity is a possibility	○	○	○	○	○
Insufficient information on the quality of functional evidence for variant classification	○	○	○	○	○
Insufficient confidence in the accuracy of functional evidence for variant classification	○	○	○	○	○
Challenges with locating functional evidence in primary literature	○	○	○	○	○
Challenges with locating functional evidence in variant databases	○	○	○	○	○

Challenges with understanding functional evidence found in primary literature	○	○	○	○	○
Challenges with understanding functional evidence found in variant databases	○	○	○	○	○
Challenges with understanding clinically relevant transcripts for hearing and/or vision	○	○	○	○	○

Please expand on any other significant challenges not described above. __________________________________

Approaches to Improving the Interaction and Application of Functional EvidenceRate what would improve your interaction with and use of functional evidence (regardless of whether it is existing or not) from 1 (not useful) to 5 (would significantly improve my interaction with and use of functional evidence).

	1 (Not useful)	2	3 (Would somewhat improve my interaction with and use of functional evidence)	4	5 (Would significantly improve my interaction with and use of functional evidence)
Workshops at professional meetings (e.g. ACMG, AMP, ASHG, ESHG, etc.) on the use of functional evidence	○	○	○	○	○
Online CEU/CME-credit training modules on using functional evidence for variant classification	○	○	○	○	○
Online non-CEU/CME-credit training modules on using functional evidence for variant classification	○	○	○	○	○
Online training spreadsheets on using functional evidence for variant classification	○	○	○	○	○
Additional VCEP-specified guidelines on the general use of functional evidence	○	○	○	○	○
Additional VCEP-specified gene-specific guidelines on the use of functional evidence	○	○	○	○	○
Additional disease-specific guidelines on the use of functional evidence	○	○	○	○	○
Improved access to primary functional data through ClinVar	○	○	○	○	○
Improved access to standardized assessments of functional data through ClinVar	○	○	○	○	○
Ability to request generation of functional data for an individual variant	○	○	○	○	○

Ability to request generation of deep mutational scanning/saturation mutagenesis/MAVE data for a specific gene (e.g. through MaveRegistry)	○	○	○	○	○

Please suggest additional ways to improve access to and use of functional evidence that was not listed above. ____

Assessment of Existing Data on Improving Clinical UtilityPlease indicate your level of agreement with the following statements about your interaction with and use of functional guidance for improving clinical utility.

	Strongly disagree	Disgree	Neither agree nor disagree	Agree	Strongly agree
The literature provides sufficient guidance demonstrating the clinical utility of utilizing variant-level functional data for variant classification.	○	○	○	○	○
The literature provides sufficient guidance demonstrating the clinical utility of utilizing variant-level functional data from MAVEs for variant classification.	○	○	○	○	○
Further clinical studies are needed to prove the clinical utility of utilizing variant-level functional data for variant classification.	○	○	○	○	○
Further clinical studies are needed to prove the clinical utility of utilizing variant-level functional data from MAVEs for variant classification.	○	○	○	○	○
When possible, clear statements about clinically relevant transcripts should be included in ClinVar and should inform MANE and/or MANE Plus Clinical isoform sets.	○	○	○	○	○

Use and Expectations of Gene-Level Functional DataPlease indicate your level of agreement with the following statements about use and expectations of gene-level functional data.

	Strongly disagree	Disgree	Neither agree nor disagree	Agree	Strongly agree
When classifying variants using gene-level data, I always read the literature that describes this functional data.	○	○	○	○	○
Gene-level data is valuable for interpreting pLOF (predicted loss-of-function) variants when variant-specific data is unavailable.	○	○	○	○	○
Gene-level experiments provide useful insights for understanding pLOF variants, even when the variants do not exactly match known cases.	○	○	○	○	○

Use and Expectations of Variant-Level Functional DataPlease indicate your level of agreement with the following statements about use and expectations of variant-level functional data.

	Strongly disagree	Disgree	Neither agree nor disagree	Agree	Strongly agree
When classifying variants using variant-level functional data, I always read the literature that describes this functional data.	○	○	○	○	○
A description of the functional assay experimental design is necessary to include in ClinVar to use variant-level functional data for variant classification.	○	○	○	○	○
If variant-level functional data is included in ClinVar, it is crucial to specify the disease mechanism to ensure appropriate use of the data for variant classification.	○	○	○	○	○
I would be more inclined to use ClinGen for variant classification if it included metrics for the confidence and accuracy of variant-level functional data in their Sequence Variant Interpretation resources.	○	○	○	○	○
I would be more inclined to use variant-level functional data for variant classification if ClinVar included links to external websites where I could explore the primary data.	○	○	○	○	○
If multiple sources of variant-level functional data are available, they should be included on the ClinVar page for a single variant, similar to how multiple variant classifications from different labs are presented.	○	○	○	○	○

If multiple conflicting sources of variant-level functional data are available, they should be included on the ClinVar page for a single variant, similar to how multiple variant classifications from different labs are presented.	○	○	○	○	○
If ClinVar included a standardized classification of variant-level functional data, such as PS3_Strong, I would utilize it for variant interpretation.	○	○	○	○	○

If there are any aspects of variant-level functional evidence in variant classification that were not addressed in this questionnaire, or if you have additional comments or suggestions, please feel free to share them here. __________________________________

Thank you for participating in our survey!

Supplementary Figures

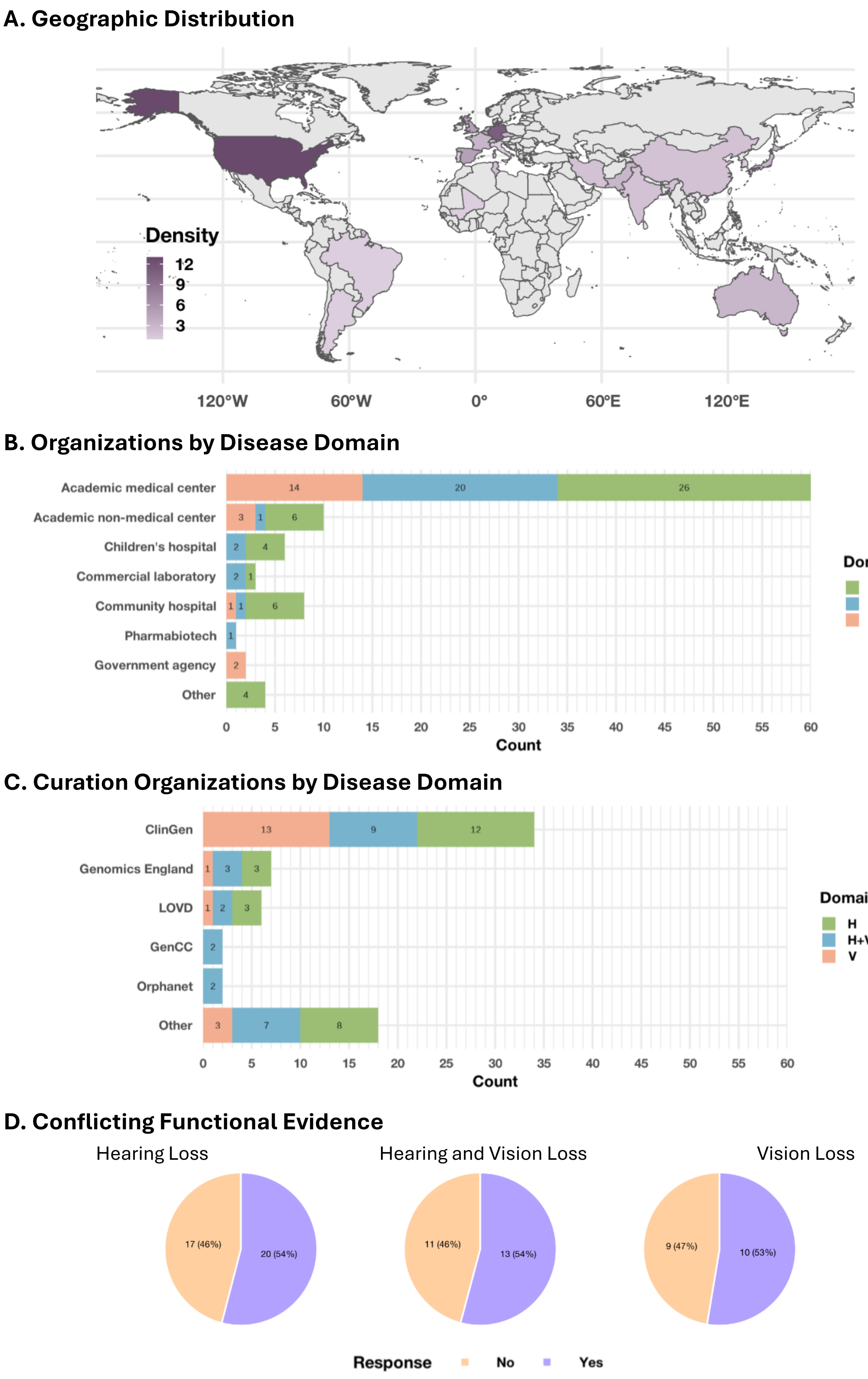
A. Geographic Distribution
Density
12
9
6
3
120°W
60°W
0°
60°E
120°E
B. Organizations by Disease Domain
Academic medical center
Academic non-medical center
Children's hospital
Commercial laboratory
Community hospital
Pharmabiotech
Government agency
Other
14
20
26
3
1
6
2
4
2
1
1
1
6
1
2
4
0
5
10
15
20
25
30
35
40
45
50
55
60
Count
Domain
H
H+V
V
C. Curation Organizations by Disease Domain
ClinGen
Genomics England
LOVD
GenCC
Orphanet
Other
13
9
12
1
3
3
1
2
3
2
2
3
7
8
0
5
10
15
20
25
30
35
40
45
50
55
60
Count
Domain
H
H+V
V
D. Conflicting Functional Evidence
Hearing Loss
Hearing and Vision Loss
Vision Loss
17 (46%)
20 (54%)
11 (46%)
13 (54%)
9 (47%)
10 (53%)
Response
No
Yes

Supplemental Figure 1. Participant characteristics and experiences with functional evidence. (**A**) Distribution of respondents by organization type. (**B**) Distribution of respondents by curation organization type. (**C**) Geographic distribution of respondents. (**D**) Proportion reporting conflicting functional evidence during variant classification.

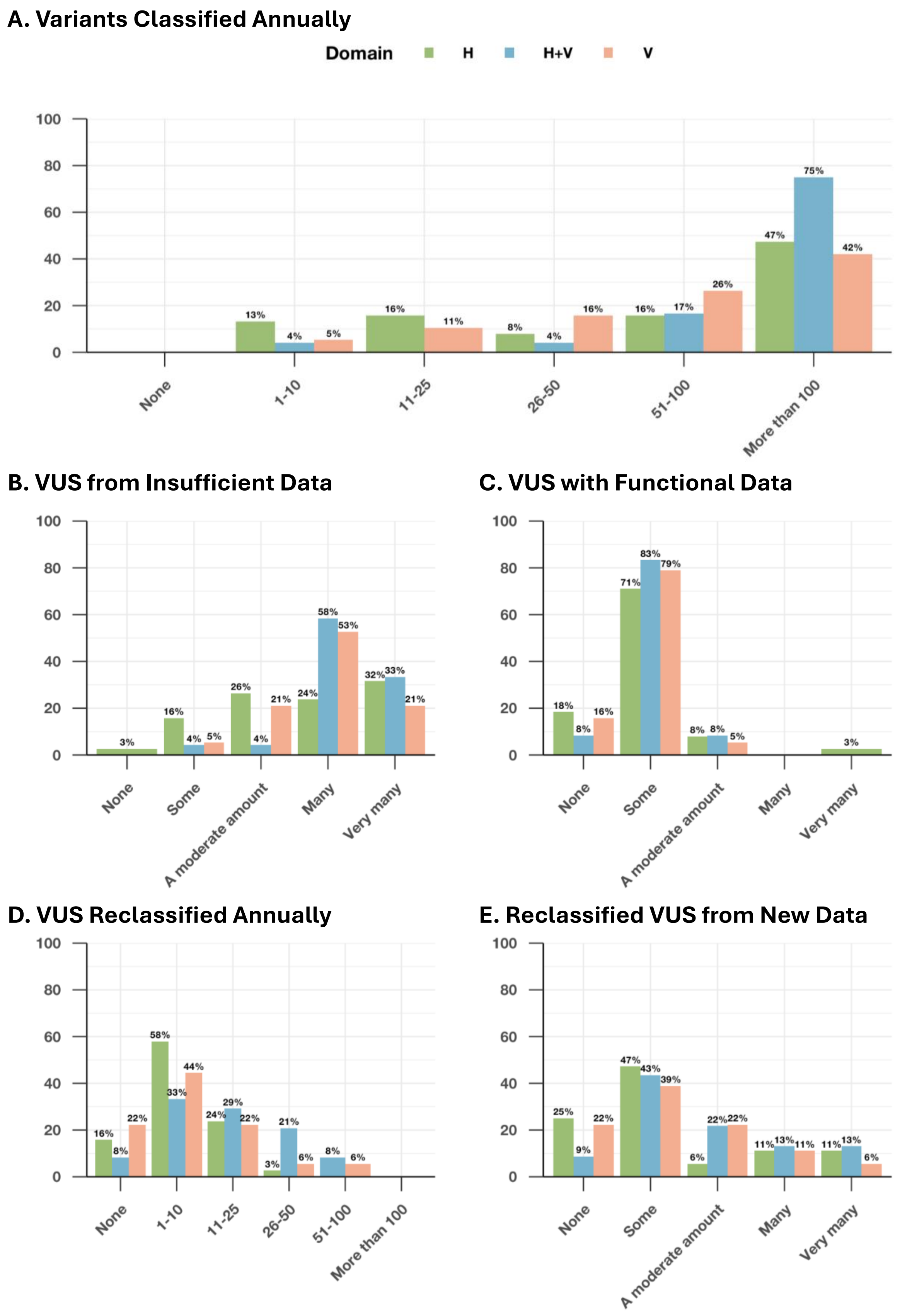

Supplemental Figure 2. Scopes and challenges of variant interpretation by disease domain. Each subplot represents responses to a different survey question: (**A**) number of variants evaluated per year, (**B**) proportion of variants classified as VUS due to insufficient data, (**C**) proportion of VUS with available functional data used for classification, (**D**) number of VUS reclassified annually, (**E**) proportion of VUS reclassified specifically due to new or updated data. Bars are grouped by disease domain: H (hearing) shown in green, H+V (hearing and vision) in blue, and V (vision) in red.

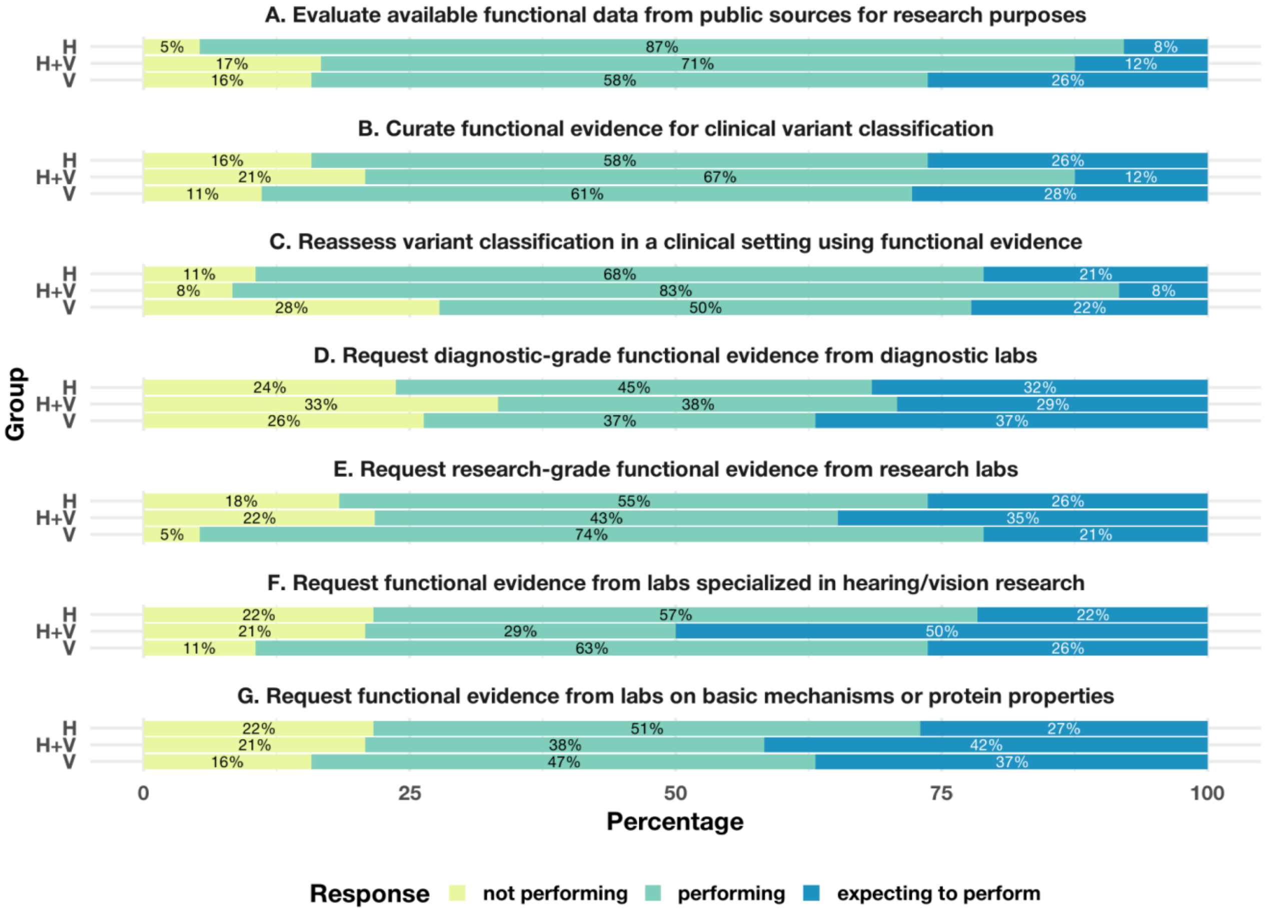

Supplemental Figure 3. Current and anticipated functional evidence tasks. Survey respondents (n = 82) (H = hearing, V = vision, H+V = both hearing and vision) reported their involvement in tasks related to the use and generation of functional evidence. Bars indicate the proportion of experts within each domain who reported not performing (yellow), currently performing (teal), or expecting to perform (blue) each task (**A**-**G**).

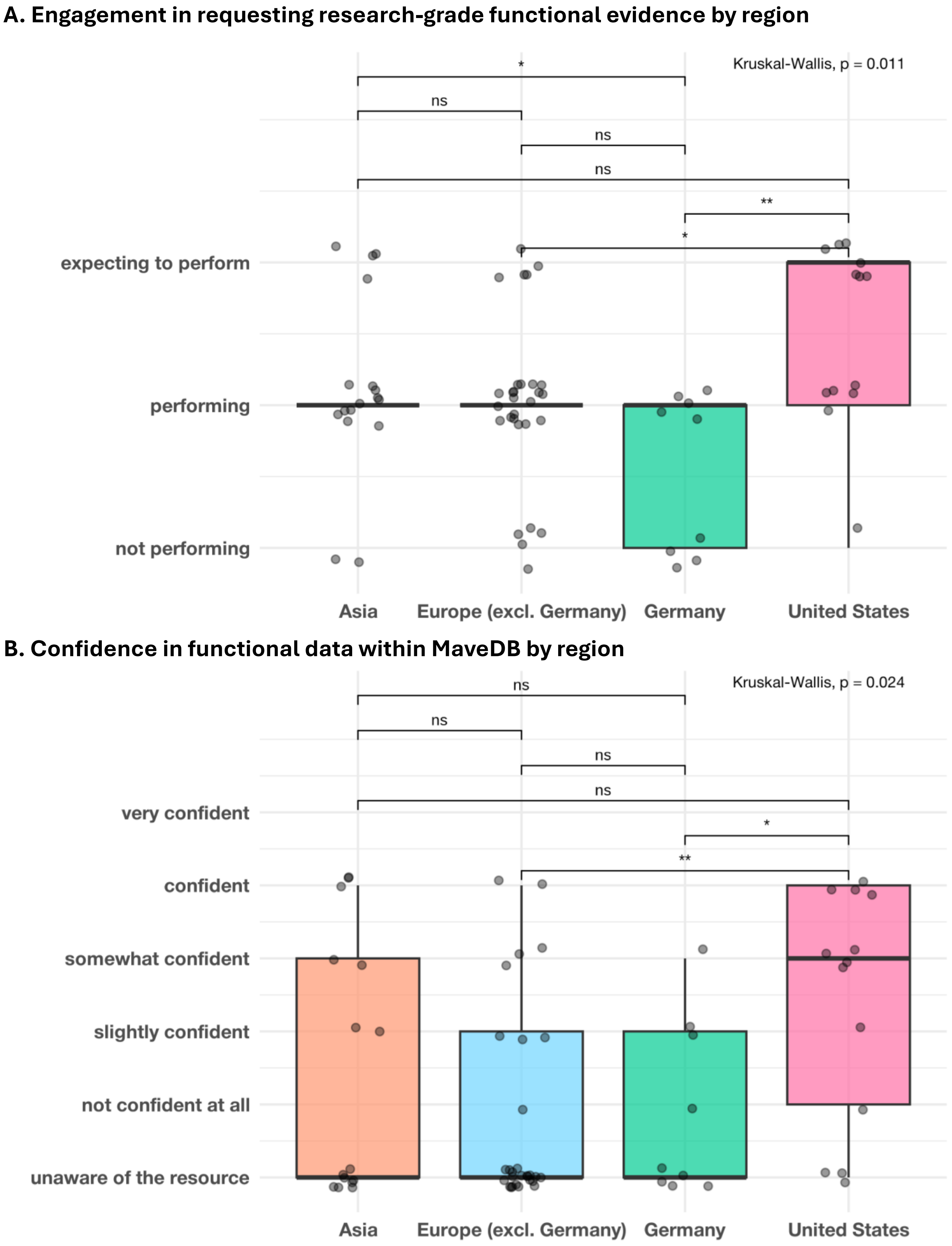

Supplemental Figure 4. Geographic patterns of confidence and engagement with functional evidence. Boxplots with jitters show (**A**) level of engagement in requesting research-grade functional evidence from research labs and (**B**) perceived confidence levels within MaveDB among hearing loss and/or vision loss experts across Asia (*n* = 17), Europe excluding Germany (*n* = 29), Germany (*n* = 11) and United States (*n* = 13). Overall differences among geographical regions were assessed using a Kruskal-Wallis test and pairwise comparisons based on Wilcoxon text were shown above each boxplot. Significance levels are indicated as follows: ns, not significant; $p \geq 0.05$; *, $p < 0.05$; **, $p < 0.01$.

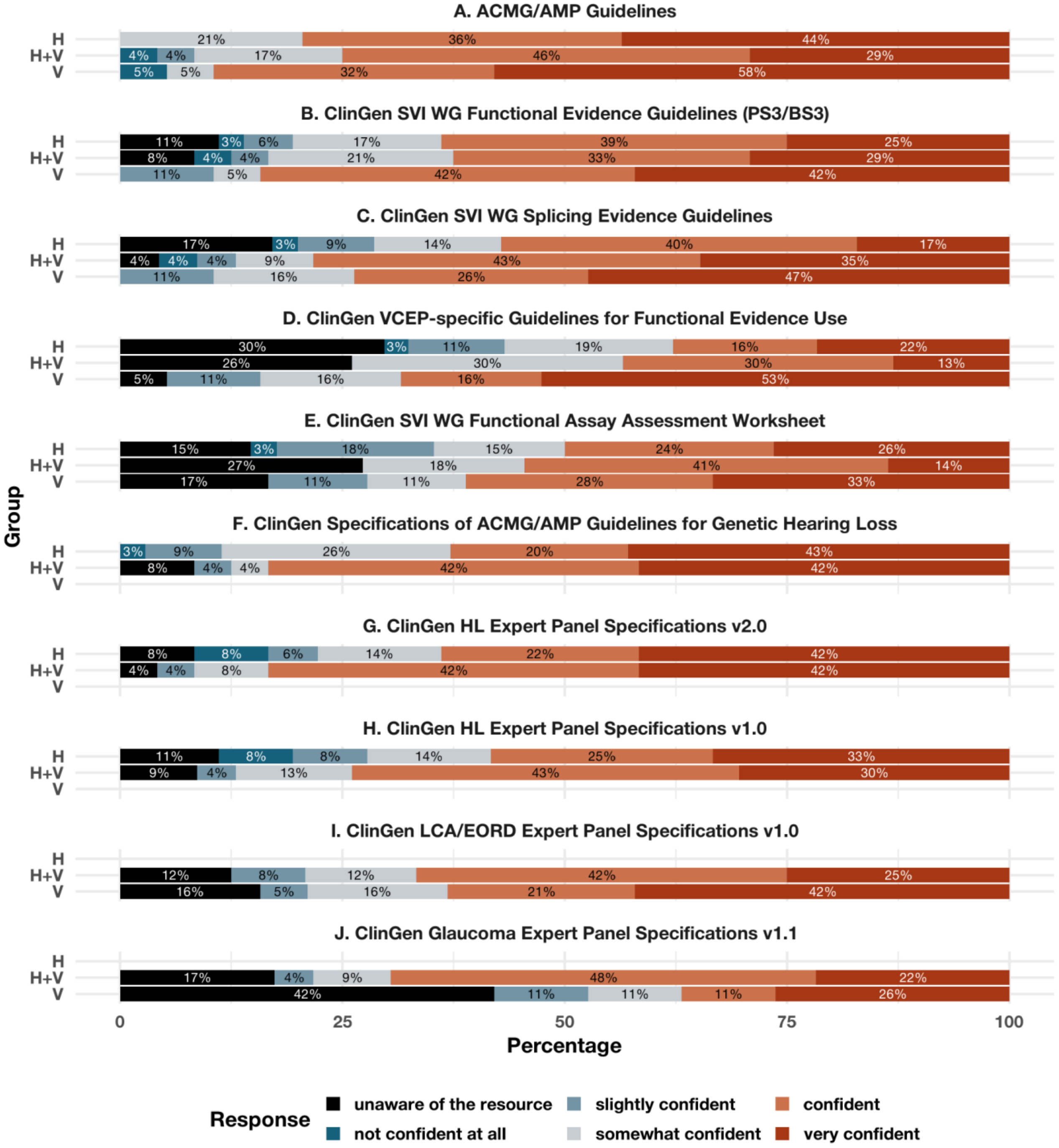

Supplemental Figure 5. Confidence in variant interpretation guidelines. Stacked bar plots display confidence levels reported in interpretation guidelines (**A**-**J**) across disease domains: hearing loss (H), ocular diseases (V), and both domains (H+V). Responses were collected on a six-point Likert scale and displayed as percentage distributions within each bar: unaware of the resource (black), not confident/slightly confident (blue), somewhat confident (grey), and (iii) confident/very confident (red). Missing responses (1-8 per item) were not shown. Abbreviations: LCA/EORD, Leber Congenital Amaurosis/early onset Retinal Dystrophy; SVI WG, Sequence Variant Interpretation Working Group.

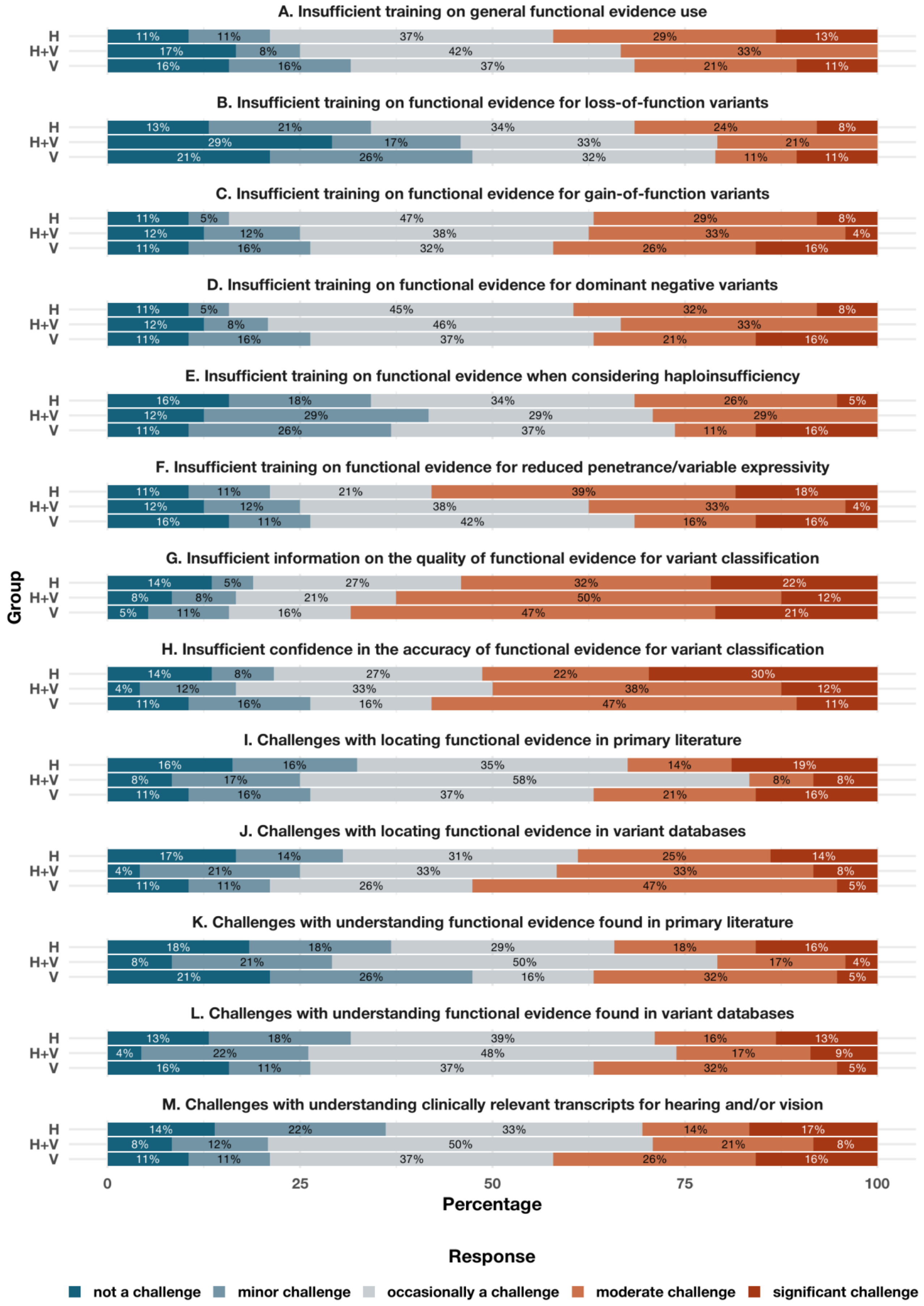

Supplemental Figure 6. Challenges in using functional evidence for variant interpretation. Stacked bar plots display

barriers to applying functional evidence (**A**-**M**) across disease domains: hearing loss (H), ocular diseases (V), and both domains (H+V). Responses were collected on a five-point Likert scale and displayed as percentage distributions within each bar: not a challenge/minor challenge (blue), occasionally a challenge (grey), and moderate challenge/significant challenge (red). Missing responses (1-3 per item) were not shown.

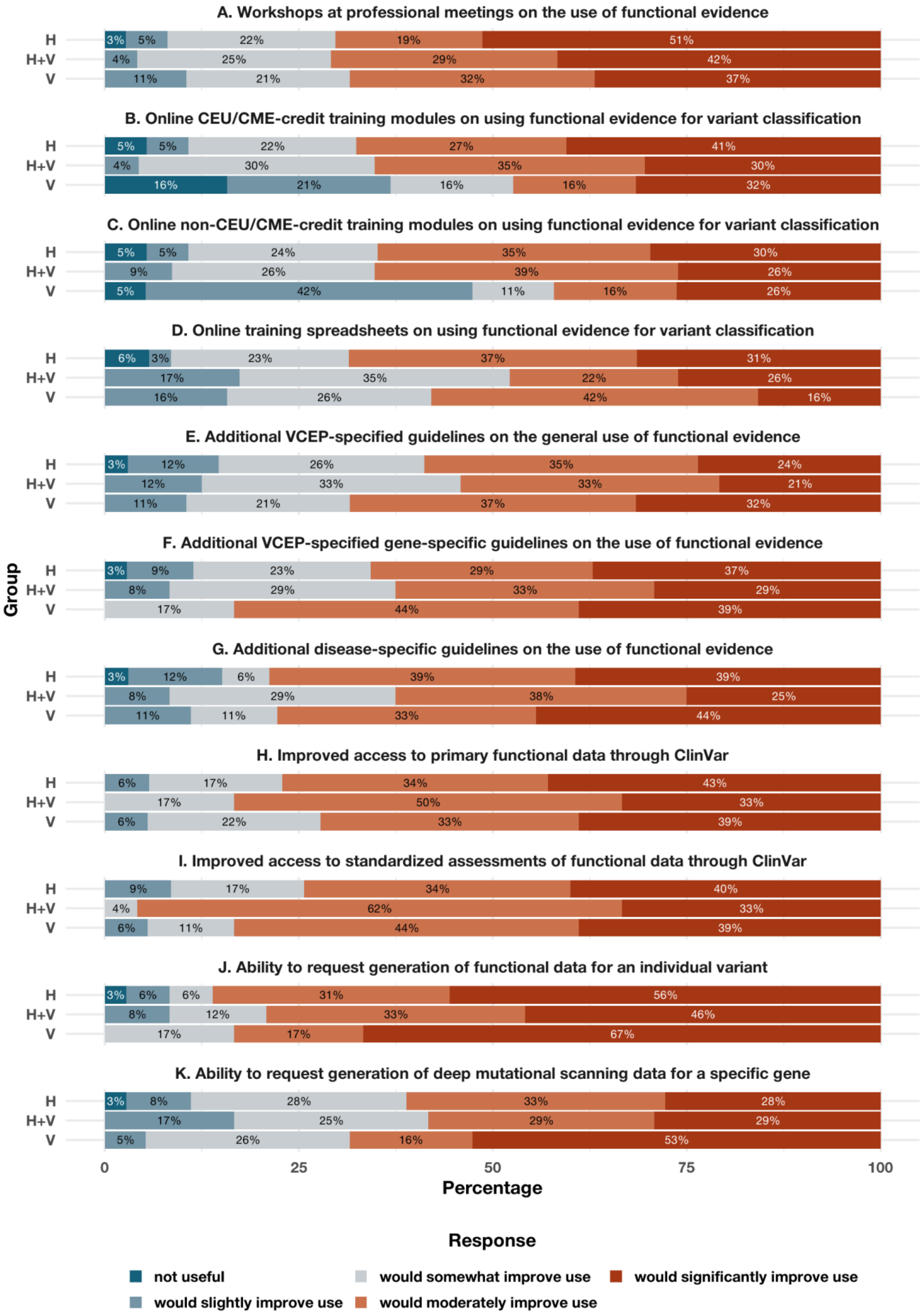
A. Workshops at professional meetings on the use of functional evidence
H 3% 5% 22% 19% 51%
H+V 4% 25% 29% 42%
V 11% 21% 32% 37%
B. Online CEU/CME-credit training modules on using functional evidence for variant classification
H 5% 5% 22% 27% 41%
H+V 4% 30% 35% 30%
V 16% 21% 16% 16% 32%
C. Online non-CEU/CME-credit training modules on using functional evidence for variant classification
H 5% 5% 24% 35% 30%
H+V 9% 26% 39% 26%
V 5% 42% 11% 16% 26%
D. Online training spreadsheets on using functional evidence for variant classification
H 6% 3% 23% 37% 31%
H+V 17% 35% 22% 26%
V 16% 26% 42% 16%
E. Additional VCEP-specified guidelines on the general use of functional evidence
H 3% 12% 26% 35% 24%
H+V 12% 33% 33% 21%
V 11% 21% 37% 32%
F. Additional VCEP-specified gene-specific guidelines on the use of functional evidence
H 3% 9% 23% 29% 37%
H+V 8% 29% 33% 29%
V 17% 44% 39%
G. Additional disease-specific guidelines on the use of functional evidence
H 3% 12% 6% 39% 39%
H+V 8% 29% 38% 25%
V 11% 11% 33% 44%
H. Improved access to primary functional data through ClinVar
H 6% 17% 34% 43%
H+V 17% 50% 33%
V 6% 22% 33% 39%
I. Improved access to standardized assessments of functional data through ClinVar
H 9% 17% 34% 40%
H+V 4% 62% 33%
V 6% 11% 44% 39%
J. Ability to request generation of functional data for an individual variant
H 3% 6% 6% 31% 56%
H+V 8% 12% 33% 46%
V 17% 17% 67%
K. Ability to request generation of deep mutational scanning data for a specific gene
H 3% 8% 28% 33% 28%
H+V 17% 25% 29% 29%
V 5% 26% 16% 53%
Group
0 25 50 75 100
Percentage
Response
not useful
would slightly improve use
would somewhat improve use
would moderately improve use
would significantly improve use

Supplemental Figure 7. Approaches to improving the interaction and application of functional evidence. Stacked bar plots display usefulness of various strategies to enhance the use of functional evidence (**A**-**K**) across disease domains: hearing loss (H), ocular diseases (V), and both domains (H+V). Responses were collected on a five-point Likert scale and displayed as percentage categories within each bar: not useful/would slightly improve use (blue), would somewhat improve use (grey), and would moderately improve use/would significantly improve use (red). Missing responses (2-7 per item) were not shown. Abbreviations: CEU, Continuing Education Units; CME, Continuing Medical Education.

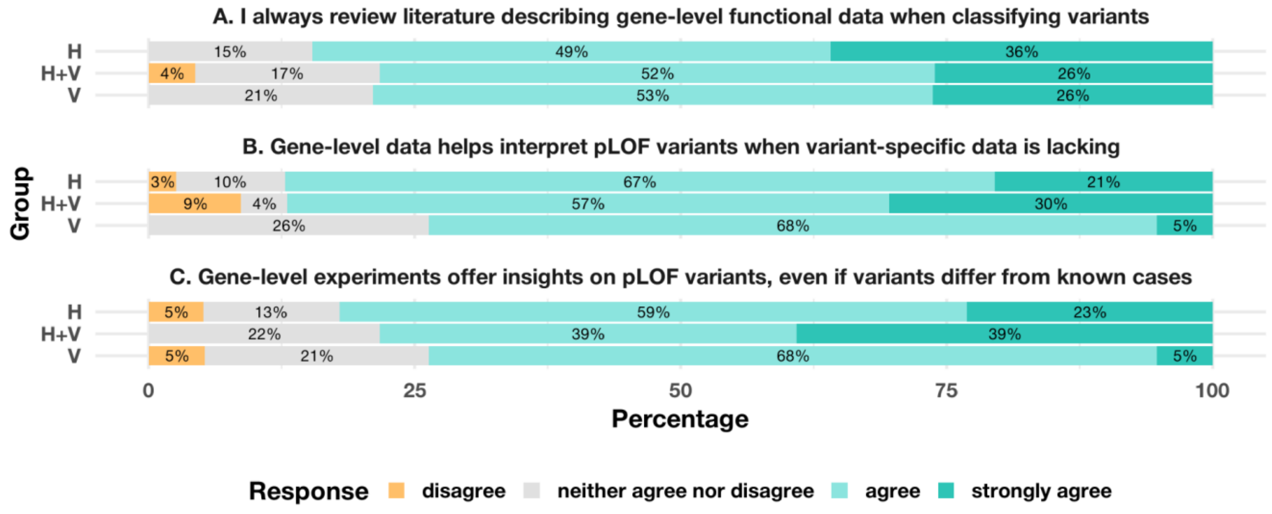

Supplemental Figure 8. Use and expectations of gene-level functional data. Stacked bar plots display agreement with statements regarding interaction with gene-level functional data (**A**-**C**) across disease domains: hearing loss (H), ocular diseases (V), and both domains (H+V). Responses were collected on a five-point Likert scale, ranging from disagree (orange) to strongly agree (cyan), with neutral responses shown in grey. Missing responses (1 per item) were not shown.

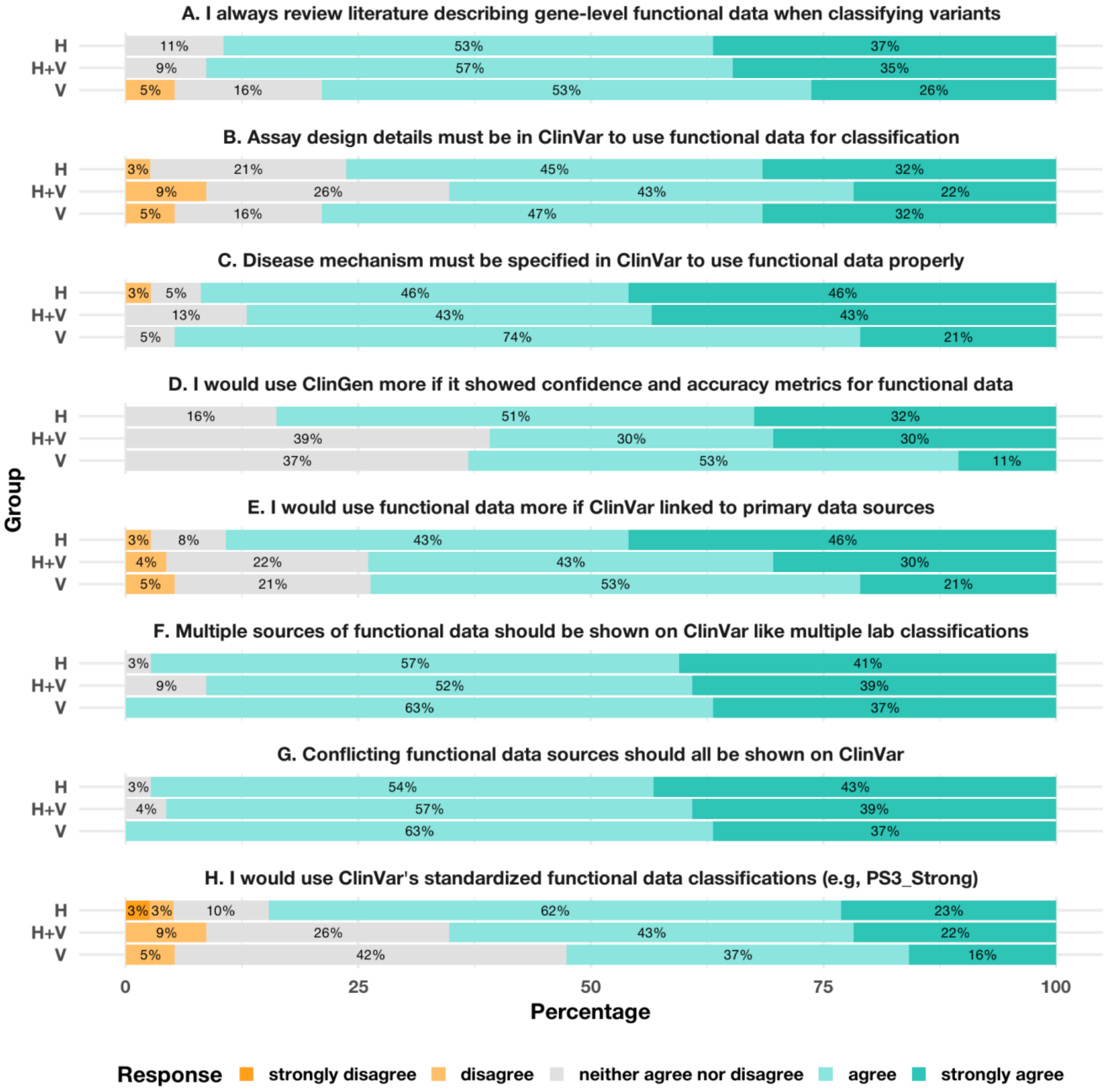

Supplemental Figure 9. Use and expectations of variant-level functional data. Stacked bar plots display agreement with statements regarding interaction with variant-level functional data (**A**-**C**) across disease domains: hearing loss (H), ocular diseases (V), and both domains (H+V). Responses were collected on a five-point Likert scale, ranging from strongly disagree (orange) to strongly agree (cyan), with neutral responses shown in grey. Missing responses (1-3 per item) were not shown.

Supplementary Table

Supplemental Table. Participant characteristics by expert group

	H (*n* = 39)	H+V (*n* = 24)	V (*n* = 19)	Total (*n* = 82)
**Sex**				
Male	36%	50%	26%	38%
Female	56%	42%	63%	54%
Not specified	8%	8%	11%	8%
**Leadership role**				
Yes	49%	62%	58%	55%
No	49%	38%	42%	44%
Not specified	2%	-	-	1%
**Professional position**				
Clinical geneticist	26%	17%	5%	18%
Genetic counselor	3%	4%	16%	6%
Laboratory director	15%	25%	11%	17%
Laboratory technician	3%	4%	-	3%
Laboratory medical geneticist	13%	21%	11%	15%
Molecular genetic pathologist	5%	-	-	2%
Ophthalmologist	-	-	5%	1%
Pathologist	2%	-	-	1%
Research scientist	26%	12%	42%	26%
Variant review scientist	2%	17%	10%	9%
Other	5%	-	-	2%
**Years of professional experience**				
0-5	31%	21%	26%	27%
6-10	13%	25%	21%	18%
11-15	18%	25%	5%	17%
16-20	7%	8%	11%	9%
More than 20	31%	21%	37%	29%

Percentages are reported within each expert group. Abbreviations: H = hearing loss experts; V = vision loss experts; H+V = cross-disciplinary experts.
