# Supplementary material for: A comparative survey of functional evidence use in hearing and vision loss genetics
Source: Commun Med (Lond). 2026 May 30;6:343. doi: 10.1038/s43856-026-01650-2 (PMC13272955; doi:10.1038/s43856-026-01650-2)
Supplement: Supplementary file 3 — Description of Additional Supplementary Files [file 43856_2026_1650_MOESM3_ESM.pdf]

Description of Additional Supplementary Files

File name: Supplementary Data 1

Description: This contains the source data for: Fig1 & SuppFig3, Fig2, Fig3, Fig4 & Supp Fig8-9, Supp Fig1A, Supp Fig1B, Supp Fig1C, Supp Fig1D, Supp Fig2A&D, Supp Fig2B&C&E, Supp Fig5, Supp Fig6, Supp Fig7

File name: Supplementary Data 2

Description: Summary of responses for engagement with functional evidence across geographic regions
