# Supplementary material for: A comparative survey of functional evidence use in hearing and vision loss genetics
Source: Commun Med (Lond). 2026 May 30;6:343. doi: 10.1038/s43856-026-01650-2 (PMC13272955; doi:10.1038/s43856-026-01650-2)
Supplement: Supplementary file 1 — Transparent Peer Review file [file 43856_2026_1650_MOESM1_ESM.pdf]

nature portfolio

Peer Review File

A Comparative Survey of Functional Evidence Use in Hearing and Vision Loss Genetics

Corresponding Author: Professor Barbara Vona

This file contains all reviewer reports in order by version, followed by all author rebuttals in order by version.

Version 0:

Reviewer comments:

Reviewer #1

(Remarks to the Author)
The authors present a comparative study on the use of functional evidence in the genetics of hearing and vision loss. This is a very comprehensive study, and the authors address many aspects related to the use of functional evidence in the classification of genetic variants associated with hearing and vision loss.
However, certain points could be developed or added in order to strengthen the study.
1-I find it inappropriate to report percentages when the total is less than 100. There are only 82 participants, and a larger sample size would provide a more representative view of the opinions of experts in auditory and visual genetics.
2-The authors indicate that 30 countries are participating in this study. Are there geographical differences in the use of functional evidence?
3- What are the real impacts of integrating functional evidence on clinical outcomes and diagnosis rates?
4-Did the authors consider conducting a longitudinal study on changes in experts' practices with regard to the integration of functional evidence?
5-The authors focus on experts, but what about patients?
6-Are there similar studies in other genetic fields, such as heart disease or cancer? If so, are the results comparable? This would help identify interesting perspectives on common and specific challenges.

Reviewer #2

(Remarks to the Author)
This research paper explores how international clinical variant curation experts in the hearing and vision loss fields utilize functional evidence to classify genetic variants and reduce the burden of variants of uncertain significance (VUS). Researchers gathered data from 82 participants to evaluate their level of trust in different types of functional evidence for variant pathogenicity, such as splicing assays, biochemical assays, animal models, and computational databases. Although the experts showed high confidence in primary literature and transcript-based studies, they expressed moderate to limited confidence in other types of evidence, such as mouse or lower-organism studies and high-throughput functional assays. Conflicting data, limited access to standardized clinical assays, and a lack of evidence-evaluation criteria were identified as important barriers to the use of functional data in variant interpretation. As a remedy, the authors advocate for improved data sharing, methodological transparency, and specialized training to enhance the clinical utility of functional evidence in variant classification. Overall, the manuscript raises important issues in the field of clinical genetics and will be of interest to the readership of your journal.
Specific comments:
1. Please describe the meaning of PS3 and BS3 when first mentioned, add a reference, and indicate whether you are still using the Richards et al. (2015) definitions (PS3: well-established in vitro or in vivo functional studies supportive of a damaging effect on the gene or gene product; BS3: well-established in vitro or in vivo functional studies showing no damaging effect on protein function or splicing), or whether any modifications have been made since then, as well as the strength level used.
2. Figure 1 is somewhat confusing. I am not sure why the axis extends to 100% on both sides and is centered on 0%. I would suggest presenting the data on a single-sided axis or as a bar graph, with the totals for each category adding up to 100%, which I think would be clearer. The same applies to similar figures (Figs. 2, 3, and Supp. Fig. 3), unless the meaning of the axis extremes is explicitly explained. For example, in Figure 2 this might represent confidence in the test, but each category

is also shown with different colours and bar widths; therefore, again, I feel that a simple bar graph would convey the same information more clearly.
3. Page 13: The “Engagement with functional evidence” section would benefit from clarification. The authors refer to diagnostic-grade functional evidence, but it is unclear what exactly this means in the context of vision and hearing loss. For example, one could imagine that a metabolic panel might serve as functional evidence for variants in genes associated with metabolic diseases. However, it is unclear what the equivalent would be in the hearing and vision loss fields; some examples and references would be helpful. I know that the authors mention such examples later in the text, but I think they should already be introduced here.
4. Pages 13–14: It would be helpful to define what the following categories actually mean:
a. public data sources (what specifically are these — MAVE databases, ClinVar, PubMed? Examples and references would be helpful);
b. reassessing variants with functional evidence (is this done through PubMed searches, unpublished sources, or other means?); and
c. curating functional data for variant classification (how does this differ from the above? What tools and data sources are used?).
5. Please make sure all abbreviations are explained in the text and figures, such as MAVE, MGI, IMPC, etc.
6. An important point that should be considered in the Discussion is that using the outcome of a functional assay for variant-effect interpretation in the context of PS3 versus BS3 is not symmetrical. A positive result in an assay (e.g., an enzymatic assay) may provide strong evidence for pathogenicity, but a negative result in the same assay is not sufficient evidence for benignity, because the variant may exert a different deleterious effect not measured by that assay (for example, a splicing defect).

Version 1:

Reviewer comments:

Reviewer #1

(Remarks to the Author)
I have no further comments to make. The authors have answered all of my questions.

Reviewer #2

(Remarks to the Author)
All my comments have been addressed. Thank you.

Open Access This Peer Review File is licensed under a Creative Commons Attribution 4.0 International License, which permits use, sharing, adaptation, distribution and reproduction in any medium or format, as long as you give appropriate credit to the original author(s) and the source, provide a link to the Creative Commons license, and indicate if changes were made.

In cases where reviewers are anonymous, credit should be given to 'Anonymous Referee' and the source.
The images or other third party material in this Peer Review File are included in the article’s Creative Commons license, unless indicated otherwise in a credit line to the material. If material is not included in the article’s Creative Commons license and your intended use is not permitted by statutory regulation or exceeds the permitted use, you will need to obtain permission directly from the copyright holder.
To view a copy of this license, visit https://creativecommons.org/licenses/by/4.0/

Point-by-Point Response to Reviewers' Comments

A Comparative Survey of Functional Evidence Use in Hearing and Vision Loss Genetics

R. Arda Inan, Marina T. DiStefano, Sami S. Amr, Tim Beißbarth, Lea M. Starita, Andrew B. Stergachis, Ahmad Abou Tayoun, Robert B. Hufnagel, Barbara Vona

Correspondence: Barbara Vona

Email: barbara.vona@med.uni-goettingen.de

Institute for Auditory Neuroscience and InnerEarLab,
University Medical Center Göttingen, Göttingen, Germany

Note: All page and line numbers refer to the clean version of the submitted manuscript.

Reviewer #1 (Remarks to the Author):

The authors present a comparative study on the use of functional evidence in the genetics of hearing and vision loss. This is a very comprehensive study, and the authors address many aspects related to the use of functional evidence in the classification of genetic variants associated with hearing and vision loss.

However, certain points could be developed or added in order to strengthen the study.

1. I find it inappropriate to report percentages when the total is less than 100. There are only 82 participants, and a larger sample size would provide a more representative view of the opinions of experts in auditory and visual genetics.

We appreciate the reviewer's concern regarding sample size. Over a six-month period, we directly contacted more than 470 individuals via email, in addition to circulating the survey through other channels. A total of 82 experts participated in our survey and we find this response rate consistent with similar studies in broader disease domains, which have recruited comparable numbers of participants:

1. Park *et al.* (2025) encompassing all disease domains recruited 190 experts
2. Villani *et al.* (2025) in Australasia included 33 participants across multiple domains
3. UK study on cancer (Allen et al., 2025) involved 46 participants.

Given the specialized nature of auditory and visual genetics, our sample size represents an acceptable proportion of the relevant expert community. As absolute counts alone could be misleading due to unequal group sizes (39 HL experts, 23 VL experts and 24 with cross-disciplinary expertise), we chose to report percentages to allow meaningful comparisons

across groups. To ensure that we do not over-interpret the results, we have made our conclusions and comparisons based on median values.

2. The authors indicate that 30 countries are participating in this study. Are there geographical differences in the use of functional evidence?

We thank the reviewer for this insightful question. Although our sample size is not sufficient to draw conclusions about geographical differences in the field-specific use of functional data, we analyzed our dataset across continents with at least 10 participants (North America (more specifically, USA), Asia and Europe) to identify trends among experts in sensory geneticists (all groups combined). This enabled us to discover two significant differences between geographical groups and we have updated the text as follows:

Page 14, line 271:
However, requests for diagnostic-grade functional evidence from clinical laboratories were relatively uncommon (37-45% vs. 43-74%) compared to research-grade evidence, likely reflecting the limited availability of approved clinical assays for both HL- and VL-associated genes (**Supplemental Figure 3D-E**). While most European experts reported sourcing research-grade functional evidence directly from laboratory studies, this practice was not widespread in the United States (**Supplemental Figure 4A**).

Page 15, Line 308
Although overall trust in MaveDB (Rubin et al., 2025) was limited across all domains (11-26%) (**Figure 3I**), the trend varied geographically as a majority of respondents from Europe (66%) and Asia (53%) were unaware of this resource (**Supplemental Table 2**, **Supplemental Figure 4B**). On the other hand, 62% of respondents from the United States reported confidence in MaveDB.

3. What are the real impacts of integrating functional evidence on clinical outcomes and diagnosis rates?

This is where MAVEs are clearly showing substantial benefit and one of the motivations for this work. There are many individual studies showing details, but for the sake of brevity, we reference Fig. 5 of the recent McEwen et al. (2026) *Nature Reviews Genetics* paper (PMID: 40691352) that summarized this impact in a single figure. This figure showed the proportion of VUS roughly being cut in half across about a dozen genes with MAVE data available. This supports that functional data are powerful for clinical variant classification away from VUS and have the potential to impact genetic diagnoses.

4. Did the authors consider conducting a longitudinal study on changes in experts' practices with regard to the integration of functional evidence?

As we collected survey responses anonymously, it is not possible for us to track changes in individual practices over time. However, we completely agree with the reviewer that a longitudinal approach could provide valuable information and we have now highlighted this as a potential direction for future research:

Page 22, line 467

A future longitudinal study could help identify how the use of functional evidence evolves in response to new assays, updated evaluation criteria and increased familiarity with available resources.

5. The authors focus on experts, but what about patients?

We thank the reviewer for this important point. The primary aim of our study was to characterize current practices, perspectives, and bottlenecks in the use of functional data among experts in hearing and ocular genetics, as variant interpretation is predominantly performed within this professional group. Establishing this baseline was particularly important given the anticipated expansion of functional datasets, including the increasing adoption of MAVEs, which are expected to substantially influence future variant interpretation workflows.

Our focus was, therefore, intentionally limited to experts to understand how functional evidence is currently generated, evaluated, and applied in clinical contexts, prior to the widespread implementation of high-throughput approaches such as MAVEs. We agree that incorporating patient perspectives is highly valuable; however, this represents a distinct and complementary research question that was beyond the scope of the present study.

We appreciate the reviewer's suggestion and agree that future work could include longitudinal assessments of how expert practices evolve over time, as well as studies exploring patient perspectives on functional data and its role in clinical decision-making.

6. Are there similar studies in other genetic fields, such as heart disease or cancer? If so, are the results comparable? This would help identify interesting perspectives on common and specific challenges.

We thank the reviewer for this suggestion. We have summarized the current literature for other fields and have drawn comparisons, where possible, in the discussion. We searched the literature for comparable studies on hearing and ocular genetics, but we did not identify field-focused studies directly analogous to our approach.

<u>Reviewer #2 (Remarks to the Author):</u>

This research paper explores how international clinical variant curation experts in the hearing and vision loss fields utilize functional evidence to classify genetic variants and reduce the burden of variants of uncertain significance (VUS). Researchers gathered data from 82 participants to evaluate their level of trust in different types of functional evidence for variant pathogenicity, such as splicing assays, biochemical assays, animal models, and computational databases. Although the experts showed high confidence in primary literature and transcript-based studies, they expressed moderate to limited confidence in other types of evidence, such as mouse or lower-organism studies and high-throughput functional assays. Conflicting data, limited access to standardized clinical assays, and a lack of evidence-evaluation criteria were identified as important barriers to the use of functional data in variant interpretation. As a remedy, the authors advocate for improved data sharing, methodological transparency, and

specialized training to enhance the clinical utility of functional evidence in variant classification. Overall, the manuscript raises important issues in the field of clinical genetics and will be of interest to the readership of your journal.

Specific comments:

1. Please describe the meaning of PS3 and BS3 when first mentioned, add a reference, and indicate whether you are still using the Richards et al. (2015) definitions (PS3: well-established in vitro or in vivo functional studies supportive of a damaging effect on the gene or gene product; BS3: well-established in vitro or in vivo functional studies showing no damaging effect on protein function or splicing), or whether any modifications have been made since then, as well as the strength level used.

> We have now updated the introduction with the statement below:
>
> Page 7, line 136:
> Specifically, we assessed the confidence of respondents in applying the ACMG/AMP Guidelines (Richards et al., 2015) and ClinGen specifications for PS3 and BS3 (Brnich et al., 2020), defined as well-established *in vitro* or *in vivo* functional studies showing a damaging or neutral effect on the gene or gene product, as well as domain-specific VCEP guidelines to evaluate functional evidence.

2. Figure 1 is somewhat confusing. I am not sure why the axis extends to 100% on both sides and is centered on 0%. I would suggest presenting the data on a single-sided axis or as a bar graph, with the totals for each category adding up to 100%, which I think would be clearer. The same applies to similar figures (Figs. 2, 3, and Supp. Fig. 3), unless the meaning of the axis extremes is explicitly explained. For example, in Figure 2 this might represent confidence in the test, but each category is also shown with different colours and bar widths; therefore, again, I feel that a simple bar graph would convey the same information more clearly.

> We thank the reviewer for the feedback regarding the figures. We have now replaced diverging bars in (Fig. 1-4 and Supp. Fig 3, 5-9) with stacked bars.

3. Page 13: The "Engagement with functional evidence" section would benefit from clarification. The authors refer to diagnostic-grade functional evidence, but it is unclear what exactly this means in the context of vision and hearing loss. For example, one could imagine that a metabolic panel might serve as functional evidence for variants in genes associated with metabolic diseases. However, it is unclear what the equivalent would be in the hearing and vision loss fields; some examples and references would be helpful. I know that the authors mention such examples later in the text, but I think they should already be introduced here.

We thank the reviewer for this helpful suggestion. Our use of the term "diagnostic-grade" refers to functional assays that are sufficiently validated, reproducible, and well-calibrated to support clinical variant interpretation under ACMG/AMP frameworks. While this may overlap with assays performed in CLIA-certified laboratories, we note that many functional studies in diagnostic labs are research-grade. Currently, there are not any diagnostic-grade functional assays for HL/VL genes.

Page 13, line 262:
Functional evidence can be broadly categorized into diagnostic-grade and research-grade evidence. Diagnostic-grade evidence refers to assays that are sufficiently validated, reproducible, and well-calibrated to support variant interpretation within ACMG/AMP frameworks.

4. Pages 13–14: It would be helpful to define what the following categories actually mean:

a. public data sources (what specifically are these — MAVE databases, ClinVar, PubMed? Examples and references would be helpful);

b. reassessing variants with functional evidence (is this done through PubMed searches, unpublished sources, or other means?); and

c. curating functional data for variant classification (how does this differ from the above? What tools and data sources are used?).

Thank you for this helpful suggestion. We have now clarified these terms in the relevant sentence as below:

Page 13, line 267:
Across all groups, most respondents reported using public data sources such as ClinVar (Landrum et al., 2014), MaveDB (Rubin et al., 2025) and PubMed (Sayers et al., 2012) (58-87%), reassessing previously classified variants by reviewing newly available functional evidence (68-83%) and curating the validity and relevance of functional assays for variant classification (58-67%) (**Supplemental Figure 3A-C**).

5. Please make sure all abbreviations are explained in the text and figures, such as MAVE, MGI, IMPC, etc.

We thank the reviewer for this helpful comment. We have ensured that all abbreviations in figures (Fig. 3, Supp. Fig. 4, Supp. Fig. 6) are explained to improve the clarity for the reader.

6. An important point that should be considered in the Discussion is that using the outcome of a functional assay for variant-effect interpretation in the context of PS3 versus BS3 is not symmetrical. A positive result in an assay (e.g., an enzymatic assay) may provide strong evidence for pathogenicity, but a negative result in the same assay is not sufficient evidence for benignity, because the variant may exert a different deleterious effect not measured by that assay (for example, a splicing defect).

We thank the reviewer for highlighting the interpretation of functional assay results under PS3 versus BS3. We have revised the Discussion to explicitly acknowledge this limitation with examples from the literature.

Page 19, line 413:
Therefore, it should be noted that functional evidence can be asymmetrical, since a damaging phenotype in a single assay does not rule out deleterious effects through mechanisms not captured by that assay (Kanavy et al., 2019). Conversely, an *in vitro* or *in vivo* damaging effect may be rescued by a *cis*-acting variant masking the clinical effect (Lee et al., 2021).

References:

Allen, S., Garrett, A., Rowlands, C. F., Durkie, M., Burghel, G. J., Robinson, R., Callaway, A., Field, J., Frugtniet, B., Palmer-Smith, S., Grant, J., Pagan, J., McDevitt, T., Snape, K., Hanson, H., McVeigh, T., Adams, D. J., Findlay, G. M., Villani, R. M., … Turnbull, C. (2025). Validating data from multiplex assays of variant effect: A CanVIG-UK national survey of NHS clinical scientists. *The American Journal of Human Genetics*, *112*(6), 1479–1488. https://doi.org/10.1016/j.ajhg.2025.04.006

Kanavy, D. M., McNulty, S. M., Jairath, M. K., Brnich, S. E., Bizon, C., Powell, B. C., & Berg, J. S. (2019). Comparative analysis of functional assay evidence use by ClinGen Variant Curation Expert Panels. *Genome Medicine*, *11*(1), 77. https://doi.org/10.1186/s13073-019-0683-1

Landrum, M. J., Lee, J. M., Riley, G. R., Jang, W., Rubinstein, W. S., Church, D. M., & Maglott, D. R. (2014). ClinVar: Public archive of relationships among sequence variation and human phenotype. *Nucleic Acids Research*, *42*(D1), D980–D985. https://doi.org/10.1093/nar/gkt1113

Lee, W., Zernant, J., Nagasaki, T., Molday, L. L., Su, P.-Y., Fishman, G. A., Tsang, S. H., Molday, R. S., & Allikmets, R. (2021). *Cis* -acting modifiers in the *ABCA4* locus contribute to the penetrance of the major disease-causing variant in Stargardt disease. *Human Molecular Genetics*, *30*(14), 1293–1304. https://doi.org/10.1093/hmg/ddab122

McEwen, A. E., Tejura, M., Fayer, S., Starita, L. M., & Fowler, D. M. (2026). Multiplexed assays of variant effect for clinical variant interpretation. *Nature Reviews Genetics*, *27*(2), 137–154. https://doi.org/10.1038/s41576-025-00870-x

On behalf of the Clinical Genome Resource Sequence Variant Interpretation Working Group, Brnich, S. E., Abou Tayoun, A. N., Couch, F. J., Cutting, G. R., Greenblatt, M. S., Heinen, C. D., Kanavy, D. M., Luo, X., McNulty, S. M., Starita, L. M., Tavtigian, S. V., Wright, M. W., Harrison, S. M., Biesecker, L. G., & Berg, J. S. (2020). Recommendations for application of the functional evidence PS3/BS3 criterion using the ACMG/AMP sequence variant interpretation framework. *Genome Medicine*, *12*(1), Article 1. https://doi.org/10.1186/s13073-019-0690-2

Oza, A. M., DiStefano, M. T., Hemphill, S. E., Cushman, B. J., Grant, A. R., Siegert, R. K., Shen, J., Chapin, A., Boczek, N. J., Schimmenti, L. A., Murry, J. B., Hasadsri, L., Nara, K., Kenna, M., Booth, K. T., Azaiez, H., Griffith, A., Avraham, K. B., Kremer, H., … ClinGen Hearing Loss Clinical Domain Working Group. (2018). Expert specification of the ACMG/AMP variant interpretation guidelines for genetic hearing loss. *Human Mutation*, *39*(11), Article 11. https://doi.org/10.1002/humu.23630

Park, M. S., Kumar, R. D., Ovadiuc, C., Folta, A., McEwen, A. E., Snyder, A., Villani, R. M., Spurdle, A. B., Fowler, D. M., Rubin, A. F., Shirts, B. H., Starita, L. M., & Stergachis, A. B. (2025). Insights on improving accessibility and usability of functional data to unlock their potential for variant interpretation. *The American Journal of Human Genetics*, *112*(6), 1468–1478. https://doi.org/10.1016/j.ajhg.2025.04.009

Richards, S., Aziz, N., Bale, S., Bick, D., Das, S., Gastier-Foster, J., Grody, W. W., Hegde, M., Lyon, E., Spector, E., Voelkerding, K., Rehm, H. L., & ACMG Laboratory Quality Assurance Committee. (2015). Standards and guidelines for the interpretation of sequence variants: A joint consensus recommendation of the American College of Medical Genetics and Genomics and the Association for Molecular Pathology. *Genetics in Medicine: Official Journal of the American College of Medical Genetics*, *17*(5), 405–424. https://doi.org/10.1038/gim.2015.30

Rubin, A. F., Stone, J., Bianchi, A. H., Capodanno, B. J., Da, E. Y., Dias, M., Esposito, D., Frazer, J., Fu, Y., Grindstaff, S. B., Harrington, M. R., Li, I., McEwen, A. E., Min, J. K., Moore, N., Moscatelli, O. G., Ong, J., Polunina, P. V., Rollins, J. E., … Fowler, D. M. (2025). MaveDB 2024: A curated community database with over seven million variant effects from multiplexed functional assays. *Genome Biology*, *26*(1), 13. https://doi.org/10.1186/s13059-025-03476-y

Sayers, E. W., Barrett, T., Benson, D. A., Bolton, E., Bryant, S. H., Canese, K., Chetvernin, V., Church, D. M., DiCuccio, M., Federhen, S., Feolo, M., Fingerman, I. M., Geer, L. Y., Helmberg, W., Kapustin, Y., Krasnov, S., Landsman, D., Lipman, D. J., Lu, Z., … Ye, J. (2012). Database resources of the National Center for Biotechnology Information. *Nucleic Acids Research*, *40*(D1), D13–D25. https://doi.org/10.1093/nar/gkr1184

Villani, R. M., Terrill, B., Tudini, E., McKenzie, M. E., Cliffe, C. C., Hahn, C. N., Lundie, B., Mattiske, T., Matotek, E., McEwen, A. E., Nickerson, S. L., Breen, J., Fowler, D. M., Christodoulou, J., Starita, L., Rubin, A. F., & Spurdle, A. B. (2025). Consultation informs strategies for improving the use of functional evidence in variant classification. *The American Journal of Human Genetics*, *112*(6), 1489–1495. https://doi.org/10.1016/j.ajhg.2025.05.003
